# A novel biocontrol agent *Bacillus velezensis* K01 for management of gray mold caused by *Botrytis cinerea*

**DOI:** 10.1186/s13568-023-01596-x

**Published:** 2023-08-29

**Authors:** Yinting Xue, Yunge Zhang, Kun Huang, Xiuyan Wang, Mingzhen Xing, Qiaolin Xu, Yanbin Guo

**Affiliations:** 1 College of Resources and Environmental Sciences, China Agricultural University, Beijing, 100193 China; 2 Hebei Innovation Center of Biofertilizer Technology, Xingtai, Hebei 054700 China

**Keywords:** *Bacillus velezensis*, Biocontrol efficiency, *Botrytis cinerea*, Antagonistic activities, Secondary metabolite

## Abstract

Gray mold is a destructive plant disease caused by a fungal pathogen *Botrytis cinerea*. The use of plant growth promoting rhizobacteria (PGPR) has proven to be a promising method to control this disease. *Bacillus velezensis* K01 was isolated from the rhizosphere of planting tomatoes. Strain K01 has a range of roles, including the ability to solubilize phytate phosphorus, stimulate resistant response, and produce indoleacetic acid (IAA), protease, cellulase, and antimicrobial substances. Strain K01 was found to inhibit 12 phytopathogenic fungi and 5 phytopathogenic bacteria. Specially, strain K01 demonstrated a biocontrol efficiency of over 78% against gray mold caused by *B. cinerea* on the leaves and fruits of tomato and pepper. Additionally, K01 was found to promote the growth of maize seedlings. Further genomic analysis revealed that K01 belongs to *B. velezensis*, which is consistent with phylogenetic analysis, average nucleotide polymorphism (ANI), and digital DNA–DNA hybridization (dDDH). The genome of strain K01 had a size of 3,927,799 bp and deduced 3866 predicted genes, with an average guanine-cytosine (GC) content of 46.5%. Based on the analyses of genomic secondary metabolites, over 18.4% of the genome was annotated to 12 gene clusters related to antimicrobial metabolite synthesis. Additionally, genome annotation and comparative genomics identified several genes associated with plant growth promotion and environmental adaption. These findings suggest that *B. velezensis* K01 has the potential to serve as a new biocontrol agent for management of gray mold on tomato and pepper.

**Supplementary Information:**

The online version contains supplementary material available at 10.1186/s13568-023-01596-x.

## Introduction

Plant diseases are one of the important reasons leading to the decrease of crop yield. According to the Food and Agriculture Organization (FAO), 14% of global crop production losses are caused by plant diseases each year, with fungal diseases accounting for 42% and bacteria for 27% (Roca-Couso et al. 2021). *Botrytis cinerea* is a necrotrophic fungus that has been reported to infect more than 1,400 plant species worldwide and causes significant economic losses of USD 10 to 100 billion over the whole growth period of the plant (Boyno et al. 2022; Nifakos et al. 2021). Control of gray mold disease caused by this fungus primarily relies on the use of chemical fungicides. However, due to the public concern for the adverse effects of fungicides on the environment and human health, applied biological control agents have emerged as a more sustainable alternative to control the plant disease in farming system, especially in organic farming system (Karthika et al. 2020; Nifakos et al. 2021). In 2021, over 76.4 million hectares of organic agricultural land were recorded in 191 countries, and the area of organic agricultural land is increased for 108.4% in past 10 years (Willer et al. 2023). The development of an effective microbial strategy comprising fungicidal properties could be a viable option for plant preharvest and postharvest health management practices in organic farming (Panneerselvam et al. 2019).

*Bacillus* is one of the most studied plant growth promoting rhizobacteria (PGPR) and has been shown to exhibit excellent capacities to promote plant growth, improve plant resistance systems, and inhibit the growth of plant pathogens (Lastochkina et al. 2019; Radhakrishnan et al. 2017; Saxena et al. 2020; Shafi et al. 2017). Species of *Bacillus* including *B. subtilis*, *B. amyloliquefaciens*, *B. methylotrophicus*, *B. polymyxa*, *B. cereus*, *B. coagulans*, *B. subterraneous*, *B. licheniformis*, *B. pumilus*, *B. circulans*, *B. velezensis*, *B. megaterium*, *B. firmus*, *B. aquimaris*, *B. vietnamensis*, and *B. aerophilus* have been reported to be suitable for biological control of plant disease and plant growth promoting (Dunlap et al. 2016; Fan et al. 2017; Saxena et al. 2020).

*B. velezensis* is a species within the genus *Bacillus*, which is widely found in soil, food, gut, and marine environments (Vairagkar et al. 2021). It has attracted significant attention due to its fast growth, harmlessness to humans and animals, and environmentally-friendly characteristics (Rabbee et al. 2019; Ye et al. 2018). *B. velezensis* was first discovered in 1998 and subsequently identified as a new species in 2016 based on comparative analysis of *B. velezensis*, *B. methylotrophicus*, and *Bacillus amylolyticus* genomes (Fan et al. 2017; Krebs et al. 1998; Ruiz-Garcia et al. 2005). Currently, *B. velezensis*, *B. amyloliquefaciens*, *Bacillus siamensis*, and *Bacillus nakamurai* are classified in the operational group of *B. amyloliquefaciens* within the Subtilis Clade of the *Bacillus* genus (Fan et al. 2017; Ngalimat et al. 2021). In 2007, the genome of the *B. velezensis* strain FZB42 was sequenced, and since then, 662 *B. velezensis* strains were whole-genome sequenced and the sequences were made available in the National Center for Biotechnology Information (NCBI) database. These reports mainly focus on analyzing and comparing the genes involved in the biosynthesis of secondary metabolites, as well as genes involved in beneficial plant-bacterial interactions in *B. velezensis* (Ngalimat et al. 2021).

Due to its abundant secondary metabolites and effects on plant growth promotion and disease inhibition, *B. velezensis* has received considerable attention in agriculture application (Saxena et al. 2020). For example, strains such as WZ-37 and BY6 could increase plant height, stem diameter, growth of tomato and poplar seedlings by produce indoleacetic acid (IAA), NH_3_, and 1-aminocyclopropane-1-carboxylate deaminase (ACC-deaminase), and strain CE 100 could decrease the incidence of *Phytophthora* root rot diseases and increase the survival rate of *Chamaecyparis obtusa* seedlings (Chen et al. 2021a; Moon et al. 2021; Zhang et al. 2021). *B. velezensis* produces a variety of secondary metabolites, such as surfactant, phonomycin, bacteriocin D, polylactic acid protein, and bacteriocin that can inhibit the development of various plant diseases caused by *Fusarium graminearum*, *B. cinerea*, *Corynespora cassiicola*, *Alternaria alternata*, and *Phytophthora capsici* on crops such as maize, grapes, cucumber, rice, chili, wheat, and watermelon (Cao et al. 2021; Chen et al. 2021b; Jiang et al. 2019; Wang et al. 2018; Xu et al. 2020). Currently, the utilization of *B. velezensis* mainly focus on promoting plant growth and biocontrol of preharvest and postharvest plant diseases. Studies on the biocontrol of postharvest diseases of plants by *B. velezensis* has been scarcely studied.

The objective of this study was to investigate the potential probiotic capabilities of the *B. velezensis* strain K01 through mining genomic sequencing information, and to determine if K01 has the potential for use as a PGPR in agriculture by conducting experiments on plant growth promotion and pathogen inhibition. Specifically, the main aims of our research were to investigate the function of K01 on (a) promoting the growth of maize, (b) antagonizing plant pathogenic fungi and controlling pepper and tomato leaf and fruit gray mold caused by *B. cinerea*, and (c) comparing and analyzing the genes associated with plant growth-promoting functions in the genome.

## Materials and methods

### Strains and culture conditions

The *B. velezensis* strain K01 was isolated from tomato rhizosphere in Weixian county, Hebei province of China, and identified as *B. velezensis* (deposited in China Center for Type Culture Collection with the deposition number of CCTCC No. M2020871). Eight plant pathogenic bacteria were incubated in Luria-Bertani (LB) medium at 28 °C. *Pseudomonas syringae*, *Pseudomonas tolaasii*, *Pectobacterium carotovorum* and *Ralstonia solanacearum* were kindly provided by Dr. Jie Feng (Plant Protection Institute, The Chinese Academy of Agricultural Sciences, Beijing, China) (Guo et al. 2007). *Agrobacterium vitis* K308 was kindly provided by Dr. Allen Kerr (Department of Plant Pathology, Waite Agricultural Research Institute, University of Adelaide, South Australia) (Kerr et al. 1980), and *Xanthomonas citri*, *Xanthomonas campestris* pv. *campestris*, and *X. campestris* pv. *vesicatoria*, were collected by our laboratory (Guo et al. 2007). Twelve pathogenic fungi were incubated in potato dextrose agar (PDA) medium at 22 °C. *B. cinerea* B02 (deposited in Agricultural Culture Collection of China with the deposition number of ACCC 35,467), *Sclerotinia sclerotiorum*, and *Colletotrichum destructivum* were kindly provided by Dr. Yue Liang (College of Plant Protection, Shenyang Agricultural University, Shenyang, China) (An et al. 2019). *Alternaria solani*, *Ceratocystis fimbriata*, *Fusarium moniliforme*, *Fusarium oxysporum*, *F. oxysporum* f. sp. *lilii, F. oxysporum* f. sp. *niveum*, *Fusarium verticillioides, Rhizoctonia cerealis*, and *Rhizoctonia solani* were kindly provided by Dr. Xili Liu (Department of Plant Pathology, China Agricultural University, Beijing, China) (Guo et al. 2007).

### Determination of antimicrobial activity in vitro

The antibacterial capacity of K01 on inhibiting the growth of the phytopathogenic bacteria was tested on LB medium according to the method of Jiao et al. (2013). The antifungal capacity of K01 on inhibiting the growth of the phytopathogenic fungi was tested on PDA medium according to the method of Liu et al. (2021).

### Biocontrol assay

The inhibition activity of K01 against grey mold caused by *B. cinerea* in tomato and pepper leaves was examined using method of Guo et al. (2007) and Chen et al. (2022) with some modifications. Five layers of sterile filter paper were spread in a petri dish (130 × 130 mm), and soaked with 10 mL sterile water. The leaves were washed three times with sterile water and placed on the moist filter paper. Bacterial cultural suspensions of K01 with 10^8^ colony-forming units (cfu mL^− 1^) was sprayed on the leaf surface. After drying the leaves, a mycelial plug of *B. cinerea* at 5 mm in diameter was placed upside down on the surface of the respective leaf. Sterile water served as a negative control and 20 mg L^− 1^ carbendazim solution was used as a positive control. At 72 h post inoculation of *B. cinerea*, the leaves were photographed with visible light and an ultraviolet lamp (365 nm; Analytik Jena US, Upland, CA, USA), and the size and area of lesions area on leaves were analyzed using the software APS Assess (APS Press, Canada). The grey mold inhibition activity was calculated as follows:

$$\text{The}\,\text{biocontrol}\,\text{efficiency}\,\text{of}\,\text{grey}\,\text{mold} \left(\%\right)=\frac{C-T}{C}\times 100\%$$

Where *C* is the average area of the lesion of the negative control group, and *T* is the average area of the lesion of leaves treated with K01.

The biocontrol activity of K01 against grey mold caused by *B. cinerea* on fruits of tomatoes, peppers, and bell peppers was analyzed according to the methods of Liu et al. (2020a) with some modifications. A 3 or 5 mm^2^ area of wound was created with a sterile needle on the surface-disinfected of tomatoes (3 mm^2^), peppers (5 mm^2^), and bell peppers (5 mm^2^) fruit. Ten microliters of K01 cultural solution with 10^8^ cfu mL^− 1^ were dropped on the wound of each fruit. A mycelial plug of *B. cinerea* with 3 or 5 mm in diameter was placed upside down on the wound of the fruit, and the fruits were cultivated at 22 °C for three days, Then, the fruits were photographed and the size of lesion area on fruits was analyzed by the method descripted above. Carbendazim solution (20 mg L^− 1^) was used as a positive control, and sterile deionized water was used as a negative control. Five replicates were set for each treatment, and the experiment was repeated three times.

### Plant growth promoting assay

The test tube maize seedling experiments according to the methods of Li et al. (2014) with some modifications. Maize caryopses were surface sterilized with 75% ethanol and germinated at 28 °C overnight. The sprouting maize caryopses were soaked in K01 bacterial suspension with at 10^6^ cfu mL^− 1^ or 10^5^ cfu mL^− 1^ for 4 h respectively. The caryopses rolled in Whatman columnar rolled filter paper, and the columnar filter paper was inserted into a tube with 25 mm of diameter and 200 mm of length. Fifteen mL Hoagland solution was added in to tubes. The tubes were incubated in a constant temperature incubator at 16 h light and 8 h dark at 22 ℃ and 70% relative humidity. There were three seedlings per test tube and five replicates per treatment. Maize caryopses were treated by sterile deionized water as the negative control. The maize samples were collected after culturing for 15 days, stem weight, and root weight were determined.

### Determination of hydrolytic activity and IAA production

Production of proteases by K01 was determined by using skim milk agar plates (Kumar et al. 2005). Cellulase was measured according to methods of Teather and Wood (1982). According to Nautiyal (1999) and Sethi and Subba-Rao (1968), the phosphate growth medium (NBRIP: glucose, 10 g; Ca_3_(PO_4_)_2_, 5 g; MgCl_2_•6H_2_O, 5 g; MgSO_4_•7H_2_O, 0.25 g; KCl, 0.2 g and (NH_4_)_2_SO_4_, 0.1 g.) of the National Institute of Botany was used to test the solubilization of tricalcium phosphate and calcium phytate activity of K01. Quantitative analysis of soluble phosphate, protease and cellulase activity were conducted according to the method of King (1932), Fischer et al. (2006), and Lynd and Zhang (2002), respectively. The production of IAA was determined using the method of Patten et al. (2002) in nutrient broth with and without tryptophan (0.5 g L^− 1^).

### Genome DNA isolation, sequencing, and the annotation

Genomic DNA of K01 was extracted using a bacterial genome isolation kit (DP302-02, Tiangen, Biotech (Beijing) Co., Ltd. Beijing, China). The whole genome of K01 was sequenced using an Illumina HiSeq 2500 system and PacBio SMRT sequencing technique (Wang et al. 2021). Subsequently, a PCR-free SMRT bell library was constructed based on the PacBio RSII/Sequel sequencing system, and the sequencing was carried out using the PacBio SMRT Technology. The hierarchical genome-assembly process method (HGAP) provided by PacBio was utilized to obtain the denovo assembly results of K01 with high integrity. The Kyoto Encyclopedia of Genes and Genomes database (KEGG, https://www.genome.jp/kegg/) and Clusters of Orthologous Groups (COG, ftp://ftp.ncbi.nlm.nih.gov/pub/COG) were employed for predicting the functional proteins (Wu et al. 2020). A circular genome map was generated using the CGview Server (http://stothard.afns.ualberta.ca/cgview_server/) to visualize gene annotation results (Grant and Stothard 2008).

### Taxonomic classification of the stain K01

A phylogenetic tree was constructed based on 16 S rRNA gene sequences given in the Online Resource (Table S1). The phylogenetic tree was generated using the maximum-likelihood method by Molecular Evolutionary Genetics Analysis version 11 (MEGA 11, Tamura et al. 2021). The average nucleotide polymorphism (ANI) values were analyzed using Jspecies WS (http://jspecies.ribohost.com/jspeciesws/), while the digital DNA–DNA hybridization (dDDH) analysis was conducted utilizing the Genome-to-Genome Distance Calculator (GGDC v3.0) (https://ggdc.dsmz.de/).

### Genes related to plant growth promotion and environmental adaption

AntiSMASH (https://antismash.secondarymetabolites.org/) was used to predict gene clusters in K01 genome related to secondary metabolism (Blin et al. 2023). Annotated gene sequences from the genome of K01 were blasted using the carbohydrate active enzyme (CAZy) database (https://bcb.unl.edu/dbCAN2/blast.php) (Zhang et al. 2022). Genes related to plant growth promoting including IAA production, phosphate solubilization, and antibiotic substance synthesis were analyzed in the K01 genome using the Basic Local Alignment Search Tool (BLAST) of NCBI (https://blast.ncbi.nlm.nih.gov/Blast.cgi).

### Statistical analysis

All data processed in the experiment were expressed as mean ± standard deviation (SD). All treatments for each experiment were compared by the *t*-test or one-way analysis of variance (ANOVA), and the post-hoc multiple comparison analysis using the Duncan multiple comparison test (*p* < 0.05) with IBM SPSS Statistics 23.0 (IBM Corp., Armonk, NY, USA). All figures were completed with Origin Pro 2021 (Origin Lab Inc., Northampton, MA, USA).

### Data availability

The complete genome sequence of *B. velezensis* K01 have been deposited in the NCBI GenBank with the accession number CP059344.1.

## Result

### The antagonistic ability of K01 strain in vitro

**Fig. 1:**
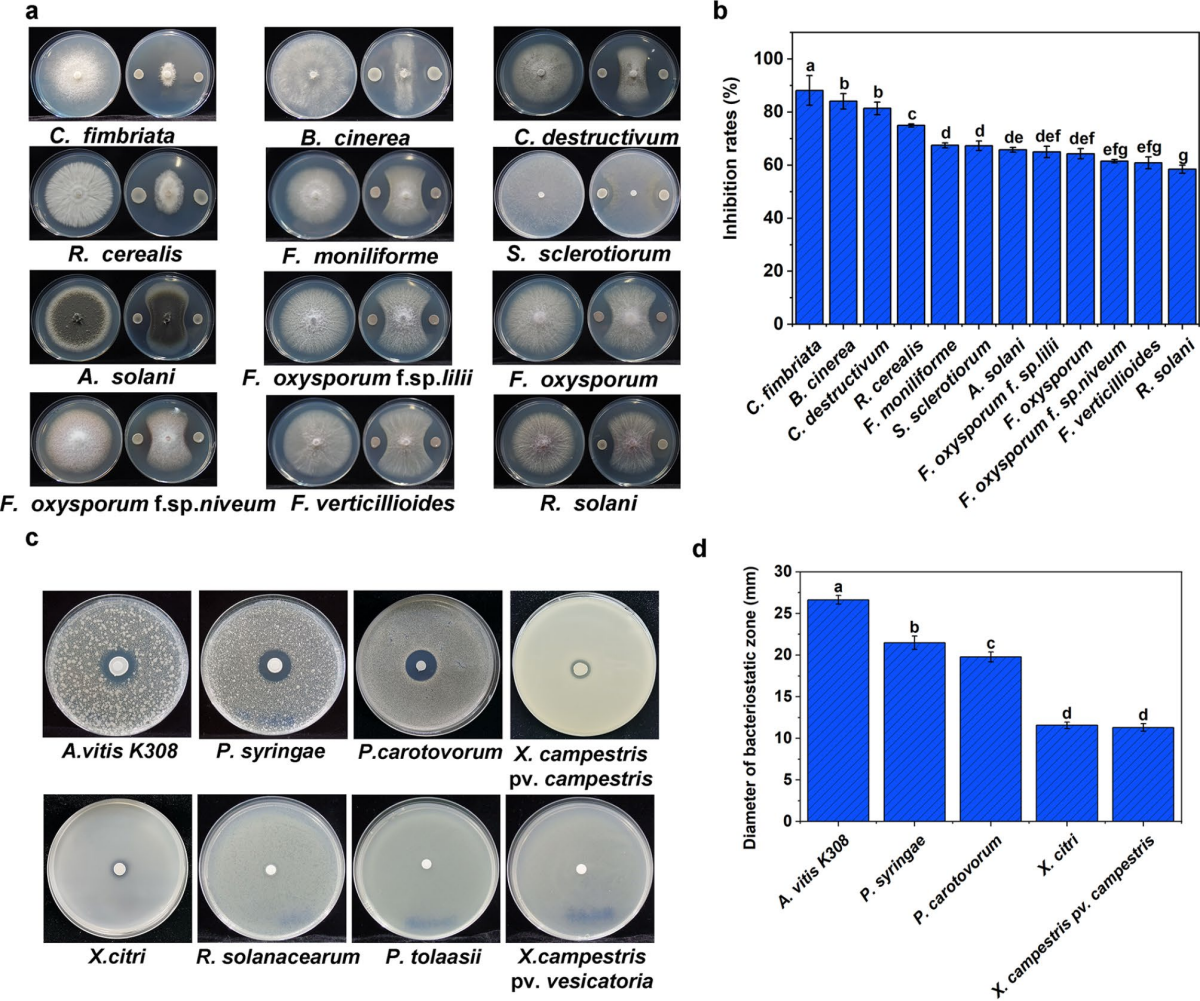
Antagonistic activity of K01 to phytopathogenic fungi **(a, b)** and phytopathogenic bacteria **(c, d)** The error bar represents the SD of the mean value of three biological repeats. Different letters above the bars indicate significant differences between treatments (one-way ANOVA, *P* < 0.05)

In this study, K01 strain was observed to display varying abilities in suppressing the growth of plant pathogenic fungi and bacteria in vitro (Fig. 1). The inhibition rate of K01 strain against 14 pathogenic fungi was more than 60%, except for *R. solani* with 58.4%. The highest inhibition to pathogenic fungi was found in *C. fimbriata* (88.1%), followed by *B. cinerea* (84.1%) and *C. destructivum* (81.4%) (Fig. 1a and b). The diameter of the inhibitory zones of K01 to pathogenic bacteria of *A. vitis*, *P. syringae*, *P. carotovorum*, *X. citri*, and *X. campestris* pv. *campestris* were 26.7, 21.5, 19.8, 11.6 and 11.3 mm, respectively (Fig. 1c and d). No antagonistic activity of K01 against *P. tolaasii, X. campestris* pv. *vesicatoria* and *R. solanacearum* were observed.

### Biocontrol gray mold on tomato and pepper by K01

**Fig. 2:**
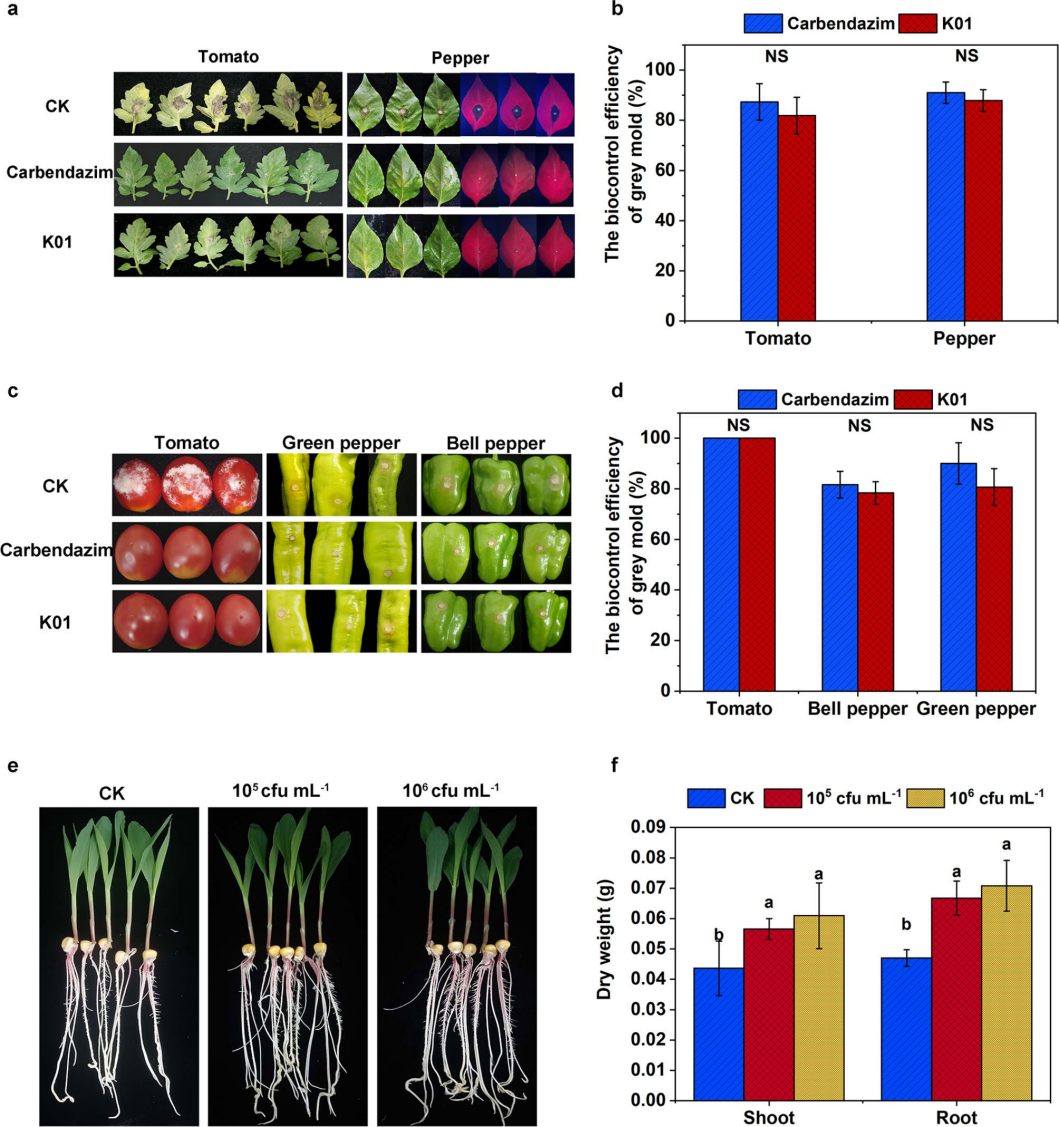
Biocontrol capability of K01 strain (**a**) K01 reduced the occurrence of gray mold in leaves, the leaves of pepper were photographed with visible light (the left three leaves) and ultraviolet lamp (the right three leaves), (**b**) The control effect of K01 on leaf gray mold in vitro, (**c**) K01 reduced the occurrence of postharvest gray mold, (**d**) Control effect of K01 on postharvest gray mold, (**e**) Growth promoting effect of K01 on maize seedlings, (**f**) Effects of K01 on stem and root dry weight of maize seedlings. The error bar represents the SD of the mean value of three biological repeats. The “NS” above the bars indicate no significant differences between treatments (t-test, *P* < 0.05), and different letters above the bars indicate significant differences between treatments (one-way ANOVA, *P* < 0.05)

The effects of strain K01 on preharvest and postharvest gray mold in tomato and pepper are shown in Fig. 2a-d. Treatment with K01 significantly reduced the lesion area caused by *B. cinerea* infection on tomato and pepper leaves (Fig. 2a). The biocontrol efficiency of K01 against gray mold on tomato and pepper leaves reached 81.9% and 87.8%, respectively (Fig. 2b). Moreover, K01 was found to be highly effective in controlling the postharvest diseases of tomato and pepper caused by *B. cinerea* (Fig. 2c). When fruits of tomato, green pepper, and bell pepper were treated with K01, the biocontrol efficiency of K01 against gray mold on the fruits reached 100%, 80.7%, and 78.4%, respectively (Fig. 2d). The biological control ability of K01 on gray mold of leaves and fruits of tomato, green pepper, and bell pepper was found to be not significantly different from that of carbendazim (Fig. 2a-d). These results indicate that K01 has the potential to be used as a biocontrol agent for effectively controlling gray mold on leaves and fruits of tomato and pepper.

### Growth promoting ability of the K01 strain

**Fig. 3:**
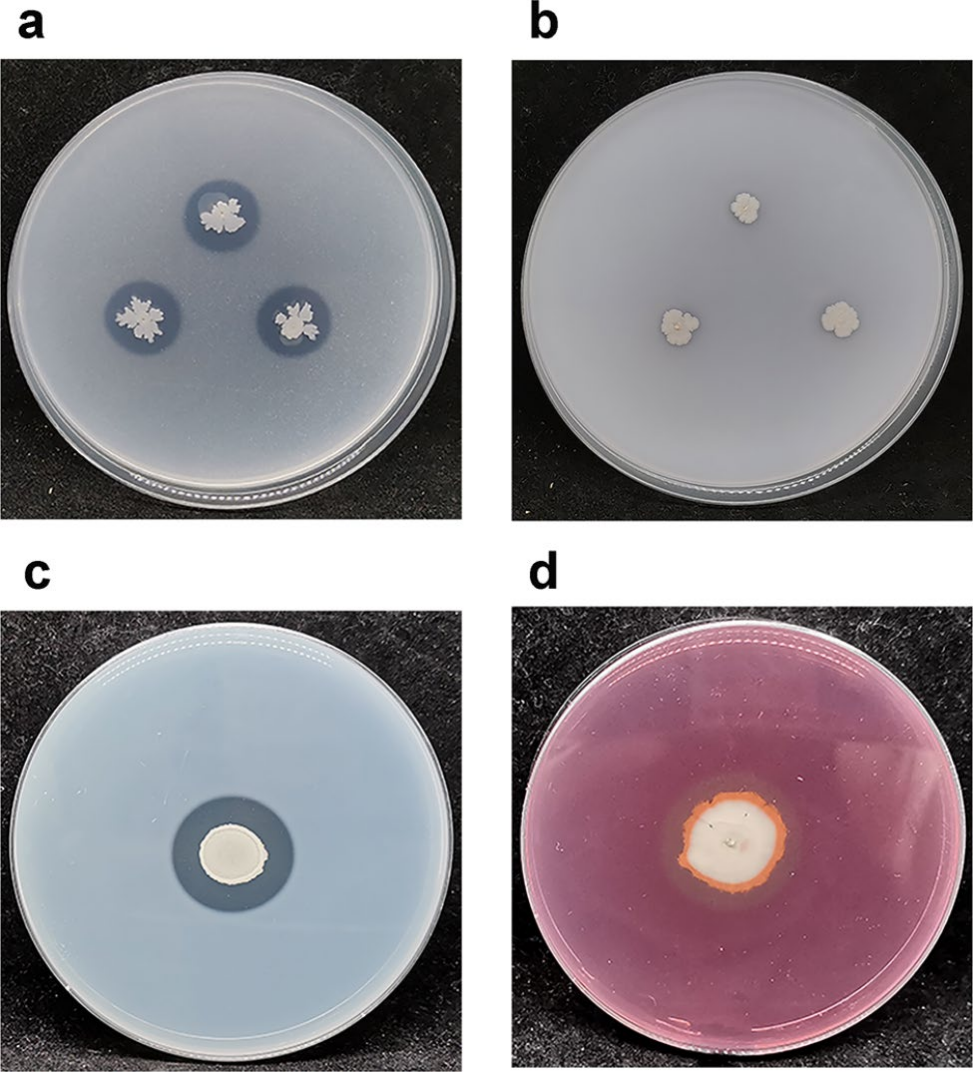
Assays for characteristics related to biocontrol (**a**) Three transparent halos were found on the plates containing phytate phosphorus, indicating that strain K01 has excellent potential for phytate phosphorus solubilization, (**b**) No transparent halo were found on the plates containing tricalcium phosphate, indicating that strain K01 may not be able to dissolve inorganic phosphorus on media containing tricalcium phosphate, (**c**) Protease production by K01 on skimmed milk agar plates, (**d**) Cellulase production by K01 on congo red cellulose plates

Upon soaking maize caryopses in K01 fermentation solution, the biomass of maize seedlings increased as compared to the control group, indicating that K01 had the capacity to promote the growth of maize seedlings (Fig. 2e-f). Treatment of maize caryopses with K01 at concentrations of 10^5^ cfu mL^− 1^ and 10^6^ cfu mL^− 1^ resulted in an increase of 29.7%, 42.0% and 39.7%, 50.6% in the dry weight of shoot and root of maize seedlings, respectively (Fig. 2f). Strain K01 has demonstrated the ability to dissolve phytate phosphorus as well as produce cellulase and protease (Fig. 3a, c, d). The content of soluble phosphorus in the media’s supernatant reached 75.3 mg/L when phytate phosphorus was supplied. The protease and cellulase activity of K01 was found to be 2683.2 and 7.6 U mL^− 1^, respectively. In addition, strain K01 produced 5.7 mg L^− 1^ IAA in DF medium supplemented with tryptophan.

### Identification of strain K01

**Fig. 4:**
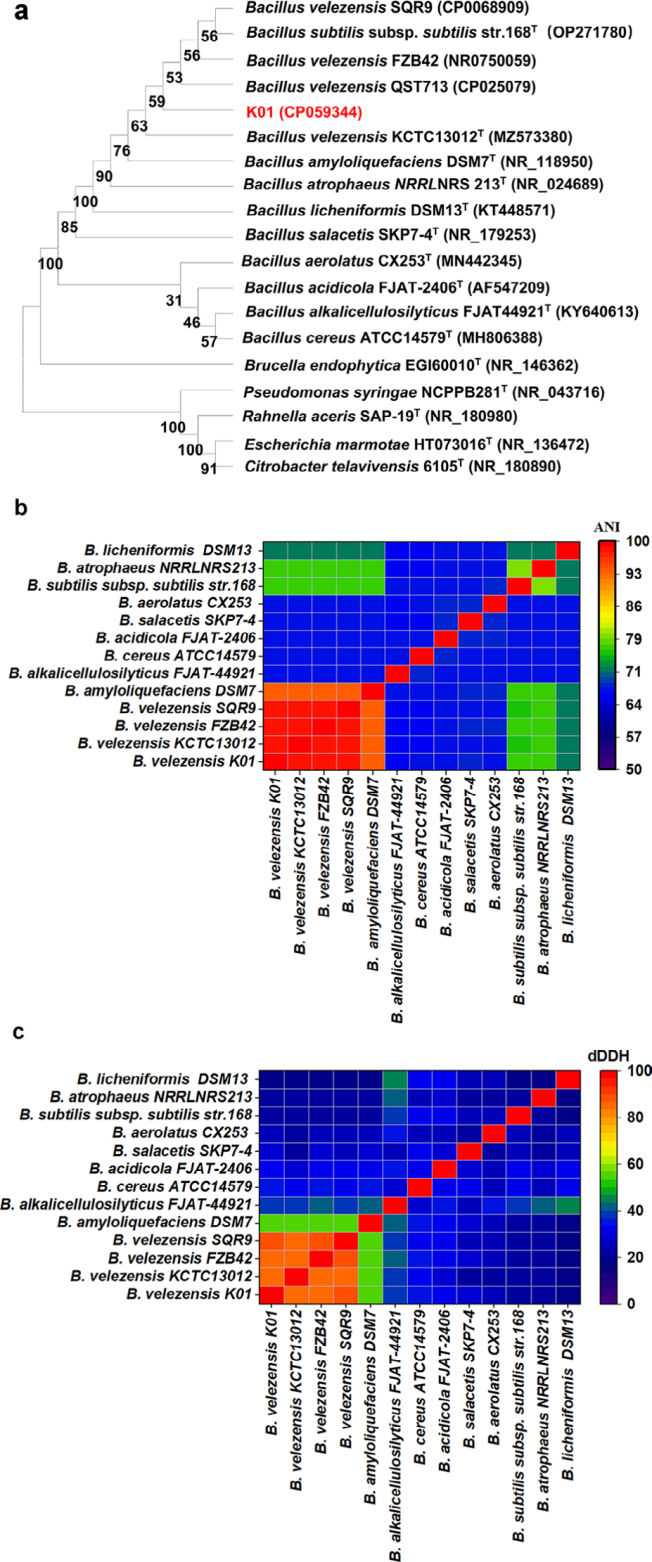
Phylogenetic classification of the strain K01 (**a**) Phylogenetic neighbor-joining tree reconstructed based on 16S rRNA genes of the selected strains of bacteria. Bootstrap values (1,000 replicates) were shown at the branch points. (**b**) ANI values were computed for a pairwise genome comparison using the Jspecies WS, and the heatmap was expressed as the percentage of ANI among the selected *Bacillus* strains. (**c**) dDDH values were calculated by using the Genome-to-Genome Distance Calculator (GGDC), and the heatmap was expressed as the percentage of dDDH among the selected *Bacillus* strains

To determine the genetic relationships of K01 with other strains in *Bacillus*, a phylogenetic tree was established based on the 16S rRNA gene sequence, and ANI and dDDH calculations among *Bacillus* strains were performed. As expected, fourteen *Bacillus* strains were clustered into two major clades. Among of them, K01 and four strains of *B. velezensis* (QST713, KCTC13012^T^, FZB42 and QST713), *B. subtilis* subsp. *subtilis* str. 168^T^, *Bacillus amyloliquefaciens* DSM7^T^, *Bacillus atrophaeus* NRRLNRS213^T^, *Bacillus licheniformis* DSM13^T^ and *Bacillus salacetis* SKP7-4^T^ were clustered into one major clade. Additional *Bacillus aerolatus* CX253^T^, *Bacillus acidicola* FJAT-2406^T^, *Bacillus cereus* ATCC14579^T^ and *Bacillus alkalicellulosilyticus* FJAT-44,921^T^ were clustered into another clade. One *Rouxiella* strain, one *Brucella* strain, one *Citrobacter* strain, one *Escherichia* strain and one *Pseudomonas* strain were in other clades (Fig. 4a). Based on the observed genetic distance relationships, strain K01 was closely clustered together with *B. velezensis* QST713, the type strain *B. velezensis* KCTC13012^T^ and *B. velezensis* FZB42, successively followed by the type strain *B. amyloliquefaciens* DSM7^T^, the type strain *B. subtilis* subsp. *subtilis* str. 168^T^ and strain *B. velezensis* SQR9 (Fig. 4a).

In the study, ANI and dDDH calculations among *Bacillus* strains were performed. The ANI values between K01 and KCTC13012^T^, FZB42 and SQR9 were 97.8%, 98.2% and 98.4%, respectively (Fig. 4b), and the dDDH values between K01 and these three strains were 84.5%, 85.4% and 89.7%, respectively (Fig. 4c). The ANI values between K01 and *B. amyloliquefaciens* DSM 7, *B. alkalicellulosilyticus* FJAT-44,921^T^, *B. cereus* ATCC14579 ^T^, *B. acidicola* FJAT-2406^T^, *B. salacetis* SKP7-4^T^, *B. aerolatus* CX253^T^, *B. subtilis* subsp. *subtilis* str.168^T^, *B. atrophaeus* NRRLNRS213^T^, and *B. licheniformis* DSM13^T^ were 93.4%, 66.7%, 66.7%,67.7%, 67.9%, 67.8%, 76.3%, 76.8%, and 71.9%, respectively (Fig. 4b), and the dDDH values between K01 and these strains were 55.5%, 38.1%, 33.7%, 30.9%, 26.8%, 25.7%, 20.9%, 20.8%, and 19.4%, respectively (Fig. 4c). Based on the results of phylogenetic tree, ANI and dDDH, strain K01 has been identified as *B. velezensis.*

### Comparative genome analysis of strain K01 with other ***B. velezensis*** strains

**Fig. 5:**
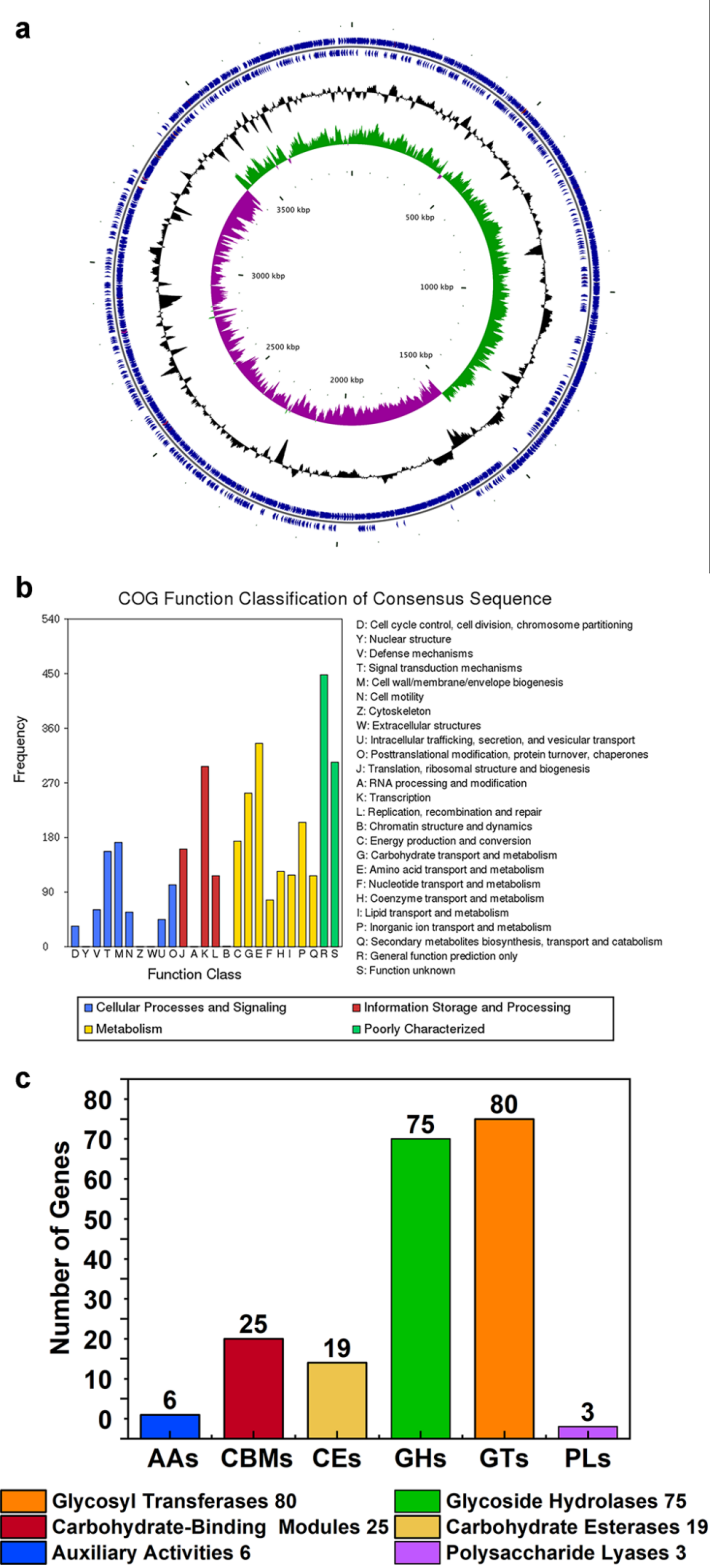
Annotation results of K01 genome and classification of CAZy gene (**a**) Circle graph: from inside to outside, the first circle is the length coordinate of genome, the second circle represents GC skew (GC SKEw = (G-C)/(G + C)), the third circle represents the average GC% content, and the outer bulge represents higher than the average and the inner concave represents lower than the average, the fourth circle was COG annotation result of negative strand gene, the fifth circle is the prediction results of negative chain genes, rRNA and tRNA, the sixth circle is the prediction results of plus-strand genes, rRNA and tRNA, the seventh circle is COG annotation result of plus-strand gene, (**b**) COG functional classification results of K01 genome, (**c**) CAZy gene classification in the K01 genome, the number on the column indicates the number of genes in that class

The genome assembly revealed that the K01 genome consisted of a single circular chromosome of 3,927,799 bp with an average GC content of 46.5%, and did not possess any plasmids (Table 1). The K01 genome was predicted to have 3,866 protein-coding genes, 86 tRNA genes, and 27 rRNA genes. The general genome structure and functions of strain K01 were illustrated in a graphical circular genome map (Fig. 5a). When comparing the genomes of *B. velezensis* K01, FZB42, and QST713, it was observed that the genome size of strain K01 was similar to FZB42 (3,918,589 bp), but smaller than that of QST713 (4,233,757 bp) (Table 1). Additionally, the average of G + C content of K01 was similar to FZB42 (46.5%) but higher than that of QST713 (45.9%) (Table 1). Moreover, the genome of K01 contained 91 pseudo genes, which was higher than the number found in FZB42 (59) and QST713 (68).

**Table 1:** The information of K01 genome and its comparison with *B. velezensis* FZB42 and QST713 strains

Properties	K01	FZB42	QST713
GenBank accession No.	CP059344.1	CP000560.2	CP025079.1
RefSeq	NZ_CP059344.1	NC_009725.2	NZ_CP025079.1
Size(bp)	3,927,799	3,918,589	4,233,757
G + C content/%	46.5	46.5	45.9
Number of genes	3,866	3,855	4,212
Protein-coding sequences (CDS)	3657	3,675	4,037
Percent of coding region (%)	94.6	95.3	95.8
Ribosomal RNA operons	27	29	25
Number of tRNAs	86	88	77
Other RNA	5	4	5
Pseudogenes	91	59	68
Bacillibactin (NRPS^a^)	50,501	98.4%	98.3%
Bacilysin (NRPS)	41,418	98.8%	98.8%
Fengycin (NRPS)	134,310	97.6%	97.5%
Bacillaene (NRPS)	100,565	98.2%	98.0%
Surfactin (NRPS)	65,407	98.2%	98.1%
Difficidin (transAT-PKS^b^)	92,359	98.0%	98.0%
Macrolactin H (transAT-PKS)	88,232	98.1%	98.0%
Butirosin A / Butirosin B (PKS-like)	41,244	98.5%	98.4%
Unknow (lanthipeptide-class-ii)	28,888	/	/
Unknow (T3PKS)	40,726	/	/
Unknow (terpene)	20,126	/	/
Unknow (terpene)	20,740	/	/

^a^ NRPS: non-ribosomal peptide synthetases, and ^b^ PKS: polyketide synthases

Of the 3,866 protein-coding genes in the K01 genome, 3,749 (97.0%) were annotated using the COG database. Among these annotated genes, 3131 were classified and annotated as being involved in amino acid transport and metabolism, transcription, carbohydrate transport and metabolism, inorganic ion transport and metabolism, energy production and conversion, and cell wall/membrane biogenesis (Fig. 5b). Within the genome, 31.3% of genes were classified under metabolism, with 219, 172, 140, 149, 69, 98, 73 and 61 genes been annotated for amino acid transport and metabolism, inorganic ion transport and metabolism, energy production and conversion, nucleotide transport and metabolism, coenzyme transport and metabolism, lipid transport and metabolism, and secondary metabolites biosynthesis transport and metabolism, respectively.

### Gene clusters involved in the synthesis of secondary metabolites

Using the AntiSMASH database, 12 putative secondary metabolite biosynthetic gene clusters (BGCs) were identified in the genome of K01, covering 18.4% (724,516 bp) of the entire genome. Of these BGCs, 5 corresponded to non-ribosomal peptide synthetases (NRPS), 2 to transAT-polyketide synthases (PKS), 2 to terpenes, 1 to PKS-like, 1 to lanthipeptide-class-ii, and 1 to T3PKS (Table 1). Most of these compounds were associated with the production of NRPS and PKS systems. Among the 12 BGCs, 8 clusters were identified to play a key role in the synthesis of bacillibactin, bacilysin, fengycin, bacillaene, surfactin, difficidin, macrolactin H and butirosin A / butirosin B (Table 1). To determine the variation of BGCs among *B. velezensis* strains and K01, their relatedness was investigated by searching for homologues all of the 8 gene clusters with *B. velezensis* FZB42 and QST713 (Table 1 and Online Resource Table S2). We identified the gene clusters responsible for synthesizing bacillibactin, bacilysin, fengycin, bacillaene, surfactin, difficidin, macrolactin H and butirosin A / butirosin B in the genome of K01. These gene clusters contain the *dhb*ABCEF, *bac*ABCDE, *fen*ABCDE, *bmy*ABCD, *srf*AAABACAD, *dfn*AYXBCDEGFHIJ, *mln*ABCDEFG and *btr*XW genes, which are 50,501 bp, 41,418 bp, 134,310 bp, 100,565 bp, 65,407 bp, 92,359 bp, 88,232 and 41,244 bp long, respectively. The amino acid sequence encoded by the gene clusters shared 98.4%, 98.8%, 97.6%, 98.2%, 98.2%, 98.0%, 98.1%, 98.5% and 98.3%, 98.8%, 97.5%, 98.0%, 98.1%, 98.0%, 98.0%, 98.4% similar with the gene cluster in *B. velezensis* FZB42 and QST713, respectively (Table 1 and Online Resource Table S2). Additionally, we identified 4 gene clusters in the genome of that encode PKS-like, lanthipeptide-class-ii, T3PKS (unknown), and terpene (unknown). These gene clusters were not found in the genomes of FZB42 and QST713.

### Analysis of CAZyme Genes in the ***B. velezensis*** K01 genome

The genome of *B. velezensis* K01 contained 208 CAZyme genes, including 6 auxiliary activity (AA) genes, 25 carbohydrate-binding module (CBM) genes, 19 carbohydrate esterase (CE) genes, 75 glycoside hydrolase (GH) genes, 80 glycosyl transferase (GT) genes, and 3 polysaccharide lyase (PL) genes (Fig. 5c and Online Resource Table S3). Lignocellulosic genes related to cellulose degradation were presented, including 15 GH13 (α-glucosidase), 2 GH5 (endo-1,4-*β*-glucanase), 2 GH30 (glucan endo-1,6-*β*-glucosidase), 5 GH4 (6-phospho-*β*-glucosidase, 6-phospho-α-glucosidase), 5 GH1 (6-phospho-*β*-galactosidase), 1 GH16 (*β*-glucanase), and 3 GH32 (two sucrose-6-phosphate hydrolase and levanase) (Online Resource Table S3). Furthermore, two cellodisaccharide hydrolase genes (GH9 and GH51) were also identified in the genome of K01. In addition, genes related to the degradation of hemicellulose (GH11, GH26, GH43, GH51, GH53, CE7), pectin (PL1and PL9), amylase (GH13, GH126, CBM34), and chitin (GH18, GH23, GT2, CE4, CBM50 and AA10) were detected in the entire genome of K01 (Online Resource Table S3).

### Genes related to plant growth promotion and environmental adaption

The draft genome of *B. velezensis* K01 was annotated to identify genes associated with plant-beneficial traits. Specifically, genes coding for plant bacteria interactions traits related to IAA synthesis were identified and annotated in genome of *B. velezensis* K01 (IAA synthesis, Online Resource Table S2). The genome was found to contain a total of 6 key genes which are involved in tryptophan synthesis (*trp*ABCDE and *TRP*1), as well as a key enzyme, aldehyde dehydrogenase (NAD+), in the pathway that produced IAA using tryptophan as a precursor. A metabolic pathway analysis using the KEGG database revealed that the genome of *B. velezensis* K01 contains 47 key genes for organic acid synthesis in glycolysis (EMP) pathway, the tricarboxylic acid cycle (TCA) pathway, the pentose phosphate pathway, and the oxidative phosphorylation pathway (Phosphorus dissolution, Online Resource Table S2). Furthermore, alkaline phosphatase coding genes, including *pho*A, *pho*D, two-component system *pho*R-*pho*P regulating phosphate synthesis and metabolism, and histidine phosphatase family protein *pho*E were also identified in the genome of K01 (Phosphorus dissolution, Online Resource Table S2). These identified genes likely play a significant role in the process of phosphate dissolution of strain K01 for promoting plant growth. Through screening and statistical analysis of protease-related genes in the whole genome of the strain, a total of 36 protease-related genes (Protease, Online Resource Table S2) were identified. These include 2 extracellular proteases *epr* and *vpr*, 6 metalloproteases *ymf*H, *ymf*F, *ymc*G, *yhf*N, *fts*H, *ywh*C, 4 serine proteases *apr*X, *htr*B, *htr*A, *yyx*A and a major intracellular serine protease, 2 cysteine proteases and 21 other proteases.

The genome of K01 was found to contain 26 genes encoding flagellar synthesis, covering the synthesis genes of the flagellar matrix, flagellar hook, and flagellar filament (Flagella synthesis, Online Resource Table S2). The flagellar collective includes *fli*F, which encodes the MS ring, *fli*E, *flg*B and *flg*C, which encode the proximal end of the matrix rod, *flg*G, which encodes the distal end of the matrix rod, *flg*H, which encodes the L ring, and *flg*I, which encodes the P ring. Additionally, the genes for the flagellar base includes *flh*A, *flh*B, *fli*H, *fli*I, *fli*K, *fli*O, *fli*Q, *fli*P and *fli*R. The genes for the flagellate hook part includes *flg*E, which encodes the flagellate hook, *flg*D, *flg*K and *flg*L, which encode proteins at the junction of the flagellate hook and the flagellate filament. The genes for the flagellar filament part includes *fli*C, which encodes the flagellar filament, as well as the flagellar cap gene *fli*D, the flagellar secretion chaperone genes *fli*S and *fli*T. The whole genome of strain K01 was found to contain genes related to chemotaxis (Chemotaxis, Online Resource Table S2), which include those for methyl-accepting chemotaxis protein (MCP), methyl transferase chemotaxis protein CheR, protein-glutamate methylesterase/glutaminase TheB (EC: 3.1.1.61 and EC: 3.5.1.44), sensor kinase TheA (EC: 2.7.13.3), purine-bond chemotactic protein TheW, chemotactic proteins TheV, TheY, TheC, TotA, TotB, flagellum motoswitch proteins FliG, FliM, and FliN, and ribose transport system substrate binding protein TbsB).

## Discussion

In recent years, the application of PGPR such as *Bacillus* has become a significant practice in agricultural production for controlling plant diseases and promoting plant growth (Shafi et al. 2017; Saxena et al. 2020). However, there are still some gaps in the specific mechanisms by which they promote plant growth and adaptation at the molecular level especially for *B. velezensis*. In this study, *B. velezensis* K01 which was isolated from tomato rhizosphere. Strain K01 produced IAA and phosphatases, which were key factors to promote plant growth, and it presented broad spectrum antagonistic activities against various plant pathogens and exhibited a significant effect in controlling *B. cinerea* on tomato and pepper. All of these characteristics indicated that K01 will be a PGPR. The complete genome of *B. velezensis* K01 was sequenced and compared with other *Bacillus* strains to better understand the mechanisms for plant-growth promotion and environmental adaption.

The phylogenetic trees showed that K01 clustered together with QST713, FZB42, KCTC13012^T^and the three strains both belonged to the *B. velezensis* species, forming a tight cluster with *B. amyloliquefaciens* DSM7^T^ and *B. subtilis* subsp. *subtilis* str. 168^T^, which was consistent with a previous study (Dunlap et al. 2016; Fan et al. 2017). Nevertheless, constructing the phylogenetic tree solely based on 16S rRNA sequences is less likely to differentiate between closely *Bacillus* species (Fan et al. 2017). Strains revealing ANI values ≥ 96% and dDDH values ≥ 70% were typically considered as the same species (Zhang et al. 2016). To more accurately determine the phylogenetic and taxonomic relationships between K01 and other *Bacillus* species, genomes must also be used to assess the similarity of closely related strains utilizing ANI and dDDH determination (Dunlap et al. 2016; Fan et al. 2017). However, our study showed that ANI and dDDH values (< 96% and < 70%, respectively) were calculated between *B. velezensis* K01 and *B. amyloliquefaciens* DSM7^T^ (93.4% and 55.5%), *B. subtilis* subsp. *Subtilis* str. 168^T^ (76.3% and 20.9%) strains (Fig. 4b-c).

In this study, strain K01 exhibited significant antagonistic effects against 12 pathogenic fungi (with inhibition rates of 58.4-88.1%) and 5 bacteria (Fig. 1). *B. velezensis* strains that have been reported to have the same ability include but not limited to FZB42, GS-1, BA-26, BS1, ZF2, BY6 and so on (Chen et al. 2007; Zhang et al. 2021, 2022; Wang et al. 2021; Shin et al. 2021; Xu et al. 2020). *B. velezensis* produces secondary metabolites against various phytopathogenic fungi and bacteria (Berezhnaya et al. 2019; Xu et al. 2020; Zhang et al. 2021). In K01 genome, 12 gene clusters related to antimicrobial compound synthesis were identified, and 8 gene clusters coding bacillibactin, bacilysin, fengycin, bacillaene, surfactin, difficidin, macrolactin H, and butirosin A / butirosin production shared 97.5–98.8% similarity to that in *B. velezensis* FZB42 and QST713. However, there were 2 gene clusters coding terpenes, and 1 gene cluster coding lanthipeptide, and 1 gene cluster coding an unknow T3PKS substance were unique identified in K01 genome compared to FZB42 and QST713. Lanthipeptides are ribosomally synthesized and post-translationally modified peptides (RiPPs) which was detected and isolated in few of bacteria with the antifungal, antibacterial, antiviral, and other activities (Repka et al. 2017). Lanthipeptide biosynthetic gene clusters were predicted in genome of *Bacillus clausii*, *B. cereus*, *Bacillus thuringiensis*, *Bacillus mycoides*, except in genome of *B. velezensis* genomes (Marsh et al. 2010, Rabbee et al. 2020). Terpenes are generally volatile, incuding α-Pinene and oxigenated terpene, which had been reported volatile antibacterial compound (De la Cruz-López et al. 2022; Stamenković et al. 2022). These metabolite synthesis gene clusters were also found in the whole genome sequence of *B. velezensis* ZF2, BIM B-439D and BY6 strains (Rabbee and Baek 2020; Xu et al. 2020; Berezhnaya et al. 2019; Zhang et al. 2021). Difficidin, macrolactin and bacilysin are primarily responsible for inhibiting pathogenic fungi, while bacillaene, fengycin and surfactin are effective against pathogenic bacteria, and bacillibactin is known to inhibit microbial competitors (Rabbee and Baek 2020). The antagonistic activity of K01 to phytopathogens was involved likely with the antimicrobial compounds predicted BGCs by antiSMASH in K01 genome.

Furthermore, we discovered that *B. velezensis* K01 has the ability to synthesize protease (Fig. 3c) and cellulase (Fig. 3d). Such activities have been reported to contribute to antagonism against phytopathogens and biocontrol of plant diseases (Xu et al. 2020). PGPRs may secrete proteases, cellulases, glucanases and chitinases to degrade components of the fungal cell wall, such as chitin, glucan, and protein, and thereby disrupt the integrity of the cell wall, ultimately reducing the pathogenicity of the phytopathogen (Huang et al. 2017). K01 strain was found to carry 5 genes related to serine proteases (Protease, Online Resource Table S2). Serine proteases are ubiquitous in the genomes of cells and viruses and employ a nucleophilic serine residue in their active sites to cleave peptides (Liu et al. 2020b). Furthermore, genes related to degradation of cellulose, hemicellulose, pectin, amylase and chitin were also detected in the genome of K01 (Online Resource Table S3). Various hydrolytic enzymes (glucanases and proteases), in turn, exhibit antifungal properties by affecting the structural elements of fungal cell walls and membranes (Lyagin et al. 2023). The endoglucanases (GH16), exo-glucanases (GH1), and β-cellobiose hydrolases (GH5) found in the genome of K01 have been identified. We therefore hypothesized that the antifungal activity of K01 might be expected to be related to the ability to decompose hydrolase activity.

Gray mold disease, caused by *B. cinerea*, can produce symptoms on all parts of vegetables, such as leaves, stem and fruits (Jiang et al. 2018; Yan et al. 2022). *B. velezensis* K01 demonstrated significant effectiveness (with biocontrol efficiency of more than 78%) in preventing and controlling preharvest and postharvest gray mold diseases in tomato and pepper (Fig. 2). The biocontrol efficiency of K01 against gray mold diseases was higher than that of *B. velezensis* A4 in tomato, strawberry, apple and kiwifruit fruits (30–70%), *B. velezensis* Bvel1 in grape fruits (14–75%), *B. velezensis* S3 and S6 in grape leaves (about 50%), and *B. velezensis* 5YN8 and DSN012 in pepper leaves (about 50%) (Calvo et al. 2020; Jiang et al. 2018; Nifakos et al. 2021; Zhao et al. 2022). *B. velezensis* has proven to be a promising and safe biocontrol agent for effectively managing gray mold disease.

Genome mining revealed that the strain K01 possesses the genetic potential to synthesize secondary metabolites, and previous studies have confirmed that several secondary metabolites can effectively control gray mold. For example, fengycin A, isolated from *B. velezensis* G341, showed in vivo antifungal activity against tomato gray mold, while iturin and different fengycin derived from *B. velezensis* BA-26 and surfactins, iturins and fengycins of strains *B. velezensis* BBC023 and BBC047 had significant inhibitory effects on *B. cinerea* (Lim et al. 2017; Salvatierra-Martinez et al. 2018; Wang et al. 2021). Indeed, several studies have highlighted that the successful use of *Bacillus* strains as biocontrol agents is not only dependent on the synthesis and secretion of various secondary metabolites with growth-promoting and antibacterial effects, but also on their ability to colonization of plants (Nifakos et al. 2021). The genome of K01 contains numerous genes responsible for flagella biosynthesis, chemotaxis, and environmental sensing receptor proteins (Online Resource Table S2), which are essential for bacterial colonization, flagella biosynthesis, and plant defense induction (Chen et al. 2007; Rabbee et al. 2019; Xu et al. 2020). These results may highlight the important potential application of K01 in agriculture.

In addition, *B. velezensis* K01 demonstrated a significant growth-promoting effect on maize. The growth-promoting effect of K01 was higher than that of strain *B. velezensis* BY6 on *Populus davidiana* seedlings (by approximately 30%) but less than that of strain *B. velezensis* BS1 on green pepper seedlings (74%) (Shin et al. 2021; Zhang et al. 2021). The reason for this phenomenon may be related to the efficiency of PGPR in synthesizing IAA. IAA production was identified in K01 cultural medium, and the IAA yield of strain K01 being less than that of *B. velezensis* T12r (48.3 mg ml^–1^) (Abdel-Hamid et al. 2021). IAA production is predominantly linked to the enzyme responsible for converting tryptophan (Abu-Zaitoon et al. 2012). Through a comparative genome analysis, we discovered that strain K01 contains the tryptophan operon *trp*ABCDE and *TRP*1, as well as the gene for an aldehyde dehydrogenase (NAD+) (IAA synthesis, Online Resource Table S2). Aldehyde dehydrogenase is involved in two IAA synthesis pathways: the tryptamine side chain oxidase pathway (TSO) and the tryptamine pathway (TAM), which oxidize indole acetaldehyde to indole acetic acid (Li et al. 2018). The synthesis pathways of IAA encoded in the strain K01 genome likely include both the TSO and the TAM pathway. Phosphorus is an essential nutrient required for plants, and its bioavailability is associated with increases in plant growth (Mpanga et al. 2020). In the natural environment, the phosphorus in soil is predominantly composed of insoluble phosphorus (Abdel-Hamid et al. 2021). Microbes can enhance phosphorus solubilization by producing organic acids, as well as by synthesizing various phosphate-solubilizing enzymes such as phytase, phosphatase, and C-P lyase (Abdel-Hamid et al. 2021; Gouda et al. 2018). Our study revealed the formation of a transparent circle on the phosphate-soluble plate (calcium phytate), indicating that strain K01 has excellent potential for phytate phosphorus solubilization (Fig. 3a). The phosphate solubilizing activity of K01 was higher than that of *B. velezensis* H11 (10.5 mg L^–1^) but lower than that of strain *B. velezensis* T12r and T13r (149.5 and 159.5 mg L^–1^) (Abdel-Hamid et al. 2021; Gutierrez-Santa Ana et al. 2020). A significant number of genes related to organic acid synthesis and alkaline phosphatase were identified in the genome of K01 strain, which may contribute to the ability of K01 to convert insoluble organic and inorganic phosphorus to soluble phosphorus (Phosphorus dissolution, Online Resource Table S2). The presence of genes related to phosphorus solubilization in the genome of K01 suggested that this strain is capable of converting both inorganic and organic phosphorus. These findings suggest that the *B. velezensis* K01 strain is endowed with PGPR traits, such as IAA production and phosphate solubilization. In the meantime, these characteristics of strain K01 may account for the growth promotion observed here in maize following inoculation.

In summary, we have shown that *B. velezensis* K01 has a broad-spectrum antagonistic activity. Additionally, it has the potential to effectively control preharvest and postharvest gray mold diseases in tomato and pepper. Based on genome sequencing information, we found 12 antimicrobial compounds synthesis gene clusters in the K01 genome, and several genes related to the synthesis of hydrolytic enzymes such as phosphatase, cellulase, and protease, which potentially are involved in the antimicrobial and plant growth promoting traits. This paper confirms evidences for *B. velezensis* K01 to serve as a PGPR and biological control agent applying in tomato, pepper, and maize production.

### Electronic supplementary material

Below is the link to the electronic supplementary material.

Supplementary Material 1

## Data Availability

Available upon the request.
