# Supplementary material for: A novel biocontrol agent Bacillus velezensis K01 for management of gray mold caused by Botrytis cinerea
Source: AMB Express. 2023 Aug 29;13:91. doi: 10.1186/s13568-023-01596-x (PMC10465465; doi:10.1186/s13568-023-01596-x)
Supplement: Supplementary file 1 — Supplementary Material 1 [file 13568_2023_1596_MOESM1_ESM.docx]

**Online Resource**

***AMB Express***

**A novel biocontrol agent *Bacillus velezensis* K01 for management gray mold caused by *Botrytis cinerea***

**Yinting Xue ^1^, Yunge Zhang ^2^, Kun Huang ^2^, Xiuyan Wang ^2^, Mingzhen Xing ^2^, Qiaolin Xu ^1^, Yanbin Guo ^1*^**

^1^ College of Resources and Environmental Sciences, China Agricultural University, Beijing 100193, China

^2^ Hebei Innovation Center of Biofertilizer Technology, Hebei Province 054700, China

*Corresponding author:

Yanbin Guo; Email: guoyb@cau.edu.cn; Tel: +86 10 6273 1457; Fax: +86 10 6273 2387

**This PDF file includes Supplementary Table S1-S3.**

**Table S1 Accession number information of the 13 strains *Bacillus* used for the average nucleotide identity (ANI) and digital DNA-DNA hybridization (dDDH) analysis in this study**

| Strains | GenBank Accession No. |
| --- | --- |
| *B. velezensis* QST713 | GCA_003073255 |
| *B. velezensis* FZB42 | GCA_000015785 |
| *B. velezensis* NRRL B-41569=CR-502=KCTC 13012^T^ | GCA_001461825 |
| *B. velezensis* SQR9 | GCA_000685725 |
| *B. amyloliquefaciens* DSM 7 = ATCC 23350 ^T^ | GCA_000196735 |
| *B. licheniformis* DSM 13 = ATCC 14580 ^T^ | GCA_000008425 |
| *B. subtilis* subsp. subtilis str. 168 ^T^ | GCA_000789275 |
| *B. atrophaeus* NRRL NRS 213=JCM 9070 ^T^ | GCA_001584335 |
| *B. acidicola* FJAT-2406= DSM 14745=105-2 ^T^ | GCA_001636425 |
| *B. alkalicellulosilyticus* FJAT-44921 ^T^ | GCA_002019795 |
| *B. aerolatus* CX253 ^T^ | GCA_009183655 |
| *B. salacetis* SKP7-4 ^T^ | GCA_003581585 |
| *B. cereus* ATCC 14579 ^T^ | GCA_000007825 |

**Table S2 Representative genes of *B. velezensis* K01 likely involved in plant growth promotion**

| Category name | Genes | Locus_Tag | Protein function |
| --- | --- | --- | --- |
| IAA synthesis | *TRP*1 | HU024_06215 | Phosphoribosylanthranilate isomerase/indole-3-glycerol phosphate synthase |
|  | *trp*E | HU024_06200 | Anthranilate synthase component I |
|  | *trp*D | HU024_06205 | Anthranilate phosphoribosyltransferase |
|  | *trp*C | HU024_06210 | Indole-3-glycerol phosphate synthase TrpC |
|  | *trp*B | HU024_06220 | Tryptophan synthase subunit beta |
|  | *trp*A | HU024_06225 | Tryptophan synthase subunit alpha |
| Soluble phosphorus | *acn*A | HU024_07710 | Aconitate hydratase AcnA |
|  | *fum*C | HU024_01370 | Class II fumarate hydratase |
|  | *mdh* | HU024_03400 | Malate dehydrogenase |
|  | gltA | HU024_11930 | Citrate synthase |
|  | *mmg*D | HU024_05395 | Citrate synthase |
|  | *icd* | HU024_03395 | NADP-dependent isocitrate dehydrogenase |
|  | *lpd*A | HU024_12630 | Dihydrolipoamide dehydrogenase |
|  | *pck*A | HU024_11930 | Phosphoenolpyruvate carboxykinase (ATP) |
|  | *pdh*A | HU024_12645 | Pyruvate dehydrogenase E1 component alpha subunit |
|  | *pdh*B | HU024_12640 | Pyruvate dehydrogenase E1 component beta subunit |
|  | *pdh*C | HU024_09385 | Pyruvate dehydrogenase complex dihydrolipoyllysine-residue acetyltransferase |
|  | *ace*F | HU024_12635 | Pyruvate dehydrogenase E2 component |
|  | *pyc* | HU024_09260 | Pyruvate carboxylase |
|  | *sdh*A | HU024_03725 | Succinate dehydrogenase flavoprotein subunit |
|  | *sdh*B | HU024_03730 | Succinate dehydrogenase iron-sulfur subunit |
|  | *sdh*C | HU024_03720 | Succinate dehydrogenase cytochrome B558 |
|  | *suc*A | HU024_07070 | 2-oxoglutarate dehydrogenase E1 component |
|  | *suc*B | HU024_07075 | 2-oxoglutarate dehydrogenase E2 component |
|  | *suc*C | HU024_08660 | ADP-forming succinate--CoA ligase subunit beta |
|  | *suc*D | HU024_08655 | Succinate--CoA ligase subunit alpha |
|  | *cel*F | HU024_17885 | 6-phospho-beta-glucosidase |
|  | PGK | HU024_00800 | Phosphoglycerate kinase |
|  | *glk* | HU024_05015 | Glucokinase |
|  | *gpm*I | HU024_00810 | 2,3-bisphosphoglycerate-independent phosphoglycerate mutase |
|  | GPI | HU024_02270 | Glucose-6-phosphate isomerase |
|  | *fbp*3 | HU024_17255 | Fructose-1,6-bisphosphatase III |
|  | *glp*X | HU024_18595 | Fructose-1,6-bisphosphatase II |
|  | GAPDH | HU024_00795 | Glyceraldehyde 3-phosphate dehydrogenase (phosphorylating) |
|  | TPI | HU024_00805 | Triose-phosphate isomerase |
|  | ENO | HU024_00815 | Phosphopyruvate hydratase |
|  | *acs*A | HU024_03060 | Acetyl-CoA synthetase |
|  | *pgm* | HU024_11995 | Phospho-sugar mutase |
|  | *fba*A | HU024_18580 | Fructose-bisphosphate aldolase |
|  | *pfk*A | HU024_03345 | 6-phosphofructokinase |
|  | *pyk* | HU024_03350 | Pyruvate kinase |
|  | *hxl*A | HU024_14870 | 3-hexulose-6-phosphate synthase |
|  | *hxl*B | HU024_14875 | 6-phospho-3-hexuloisomerase |
|  | PRPS | HU024_16375 | Ribose-phosphate diphosphokinase |
|  | *rpi*B | HU024_18680 | Ribose 5-phosphate isomerase B |
|  | *gnd*A | HU024_05525 | NADP-dependent phosphogluconate dehydrogenase |
|  | *zwf* | HU024_05530 | Glucose-6-phosphate dehydrogenase |
|  | *eda* | HU024_07630 | 2-keto-3-deoxy-6-phosphogluconate aldolase |
|  | *deo*C | HU024_17500 | Deoxyribose-phosphate aldolase |
|  | *fsa* | HU024_18585 | Fructose-6-phosphate aldolase |
|  | *deo*B | HU024_05810 | Phosphopentomutase |
|  | *ppa*X | HU024_00315 | Pyrophosphatase PpaX |
|  | *ppa*C | HU024_16945 | Manganese-dependent inorganic pyrophosphatase |
|  | *pho*E | HU024_11505 | Histidine phosphatase family protein |
|  | *pho*A | HU024_11945 | Alkaline phosphatase |
|  | *pho*D | HU024_15225 | Alkaline phosphatase |
|  | *pho*P | HU024_03405 | Alkaline phosphatase synthesis response regulator PhoP |
|  | *pho*R | HU024_03410 | Phosphate regulon sensor histidine kinase PhoR |
|  | *pho*H | HU024_04785 | PhoH family protein |
|  | *pho*H2 | HU024_09285 | PhoH family protein |
| Protease | *ctpB* | HU024_00200 | Carboxy-terminal processing protease CtpB |
|  | *clpP* | HU024_00505 | ATP-dependent Clp protease proteolytic subunit |
|  | *paiA* | HU024_00570 | Protease synthase and sporulation negative regulatory protein paiA |
|  | *htrB* | HU024_01395 | Serine protease Do-like HtrB |
|  | *YugP* | HU024_02300 | Membrane protease YugP |
|  |  | HU024_02355 | Cysteine protease YraA |
|  |  | HU024_03100 | Cysteine protease YraA |
|  | *clpX* | HU024_03840 | ATP-dependent Clp protease ATP-binding subunit ClpX |
|  | *lonB* | HU024_03845 | Lon protease 2 |
|  | *lon* | HU024_03850 | Lon protease 1 |
|  | *yrrN* | HU024_04265 | Uncharacterized protease YrrN |
|  | *yrrO* | HU024_04270 | Uncharacterized protease YrrO |
|  | *gpr* | HU024_04685 | Germination protease |
|  | *prsW* | HU024_06070 | Protease PrsW |
|  | *ctpA* | HU024_06940 | Carboxy-terminal processing protease CtpA |
|  | *aprX* | HU024_08080 | Serine protease AprX |
|  | *ymfH* | HU024_08265 | Uncharacterized zinc protease YmfH |
|  | *ymfF* | HU024_08270 | Inactive metalloprotease YmfF |
|  | *ymxG* | HU024_08345 | Uncharacterized zinc protease YmxG |
|  | *rseP* | HU024_08420 | Regulator of sigma-W protease RasP |
|  | *hslU* | HU024_08625 | HslU--HslV peptidase ATPase subunit |
|  | *hslV* | HU024_08630 | ATP-dependent protease subunit HslV |
|  | *clpE* | HU024_09875 | ATP-dependent Clp protease ATP-binding subunit ClpE |
|  | *htpX* | HU024_09975 | Protease HtpX homolog |
|  |  | HU024_10080 | Major intracellular serine protease |
|  | *htrA* | HU024_10225 | Serine protease Do-like HtrA |
|  | *yhfN* | HU024_11520 | Uncharacterized metalloprotease YhfN |
|  |  | HU024_12480 | Serine protease |
|  | *paiB* | HU024_13865 | Protease synthase and sporulation protein paiB |
|  | *YdcA* | HU024_14235 | Rhomboid protease YdcA |
|  | *ftsH* | HU024_16275 | ATP-dependent zinc metalloprotease FtsH |
|  | *yabG* | HU024_16415 | Sporulation-specific protease YabG |
|  | *yyxA* | HU024_17090 | Uncharacterized serine protease YyxA |
|  | *epr* | HU024_17935 | Minor extracellular protease Epr |
|  | *vpr* | HU024_18095 | Minor extracellular protease vpr |
|  | *ywhC* | HU024_18420 | Zinc metalloprotease YwhC |
| Flagellar synthesis | *flg*M | HU024_00100 | Flagellar biosynthesis anti-sigma factor FlgM |
|  | *flg*K | HU024_00110 | Flagellar hook-associated protein FlgK |
|  | *flg*L | HU024_00115 | Flagellar hook-associated protein FlgL |
|  | *fli*C | HU024_00135 | Flagellin |
|  | *fli*D | HU024_00140 | Flagellar hook-associated protein 2 |
|  | *fli*S | HU024_00145 | Flagellar export chaperone FliS |
|  | *fli*T | HU024_00150 | Flagella biosynthesis regulatory protein FliT |
|  | *flh*A | HU024_08505 | Flagellar biosynthesis protein FlhA |
|  | *flh*B | HU024_08510 | Flagellar biosynthesis protein FlhB |
|  | *fli*R | HU024_08515 | Flagellar type III secretion system protein FliR |
|  | *fli*Q | HU024_08520 | Flagellar biosynthesis protein FliQ |
|  | *fli*P | HU024_08525 | Flagellar type III secretion system pore protein FliP |
|  | *fli*O,*fli*Z | HU024_08530 | Flagellar protein FliO/FliZ |
|  | *fli*L | HU024_08550 | Flagellar protein FliL |
|  | *flg*D | HU024_08565 | Flagellar hook assembly protein FlgD |
|  | *fli*K | HU024_08570 | Flagellar hook-length control protein FliK |
|  | *fli*J | HU024_08580 | Flagellar biosynthesis chaperone FliJ |
|  | *fli*I | HU024_08585 | Flagellar protein export ATPase FliI |
|  | *fli*H | HU024_08590 | Flagellar assembly protein FliH |
|  | *fli*F | HU024_08600 | Flagellar M-ring protein FliF |
|  | *fli*E | HU024_08605 | Flagellar hook-basal body complex protein FliE |
|  | *flg*C | HU024_08610 | Flagellar basal body rod protein FlgC |
|  | *flg*B | HU024_08615 | Flagellar basal body rod protein FlgB |
|  | *flg*E | HU024_18935 | Flagellar hook-basal body protein |
| Chemotaxis | *mcp* | HU024_02335 | Methyl-accepting chemotaxis protein |
|  | *che*D | HU024_08470 | Chemotaxis protein CheD |
|  | *che*R | HU024_06180 | Chemotaxis protein methyltransferase CheR |
|  | *che*B | HU024_08490 | Two-component system, chemotaxis family, protein-glutamate methylesterase/glutaminase |
|  | *che*A | HU024_08485 | Two-component system, chemotaxis family, sensor kinase CheA |
|  | *che*W | HU024_08480 | Purine-binding chemotaxis protein CheW |
|  | *che*V | HU024_08535 | Two-component system, chemotaxis family, chemotaxis protein CheV |
|  | *che*Y | HU024_09710 | Two-component system, chemotaxis family, chemotaxis protein CheY |
|  | *che*C | HU024_08475 | Chemotaxis protein CheC |
|  | *fli*G | HU024_08595 | Flagellar motor switch protein FliG |
|  | *fli*M | HU024_08545 | Flagellar motor switch protein FliM |
|  | *fli*N | HU024_08540 | Flagellar motor switch protein FliN |
|  | *mot*A | HU024_09880 | Chemotaxis protein MotA |
|  | *mot*B | HU024_09885 | Chemotaxis protein MotB |
|  | *rbs*B | HU024_19150 | Ribose ABC transporter substrate-binding protein RbsB |
| Bacillibactin synthesis | *dhb*A | HU024_01945 | 2,3-dihydro-2,3-dihydroxybenzoate dehydrogenase |
|  | *dhb*C | HU024_01950 | Isochorismate synthase DhbC |
|  | *dhb*E | HU024_01955 | (2,3-dihydroxybenzoyl) adenylate synthase |
|  | *dhb*B | HU024_01960 | Isochorismatase |
|  | *dhb*F | HU024_01965 | Non-ribosomal peptide synthetase |
| Difficidin synthesis | *dfn*A | HU024_05570 | ACP S-malonyltransferase |
|  | *dfn*Y | HU024_05575 | D-fructose-6-phosphate amidotransferase |
|  | *dfn*X | HU024_05580 | Acyl carrier protein |
|  | *dfn*B | HU024_05585 | Long-chain fatty acid--CoA ligase |
|  | *dfn*C | HU024_05590 | SDR family oxidoreductase |
|  | *dfn*D | HU024_05595 | SDR family NAD(P)-dependent oxidoreductase |
|  | *dfn*E | HU024_05600 | KR domain-containing protein |
|  | *dfn*F | HU024_05605 | Type I polyketide synthase |
|  | *dfn*G | HU024_05610 | SDR family NAD(P)-dependent oxidoreductase |
|  | *dfn*H | HU024_05615 | SDR family NAD(P)-dependent oxidoreductase |
|  | *dfn*I | HU024_05620 | Type I polyketide synthase |
|  | *dfn*J | HU024_05625 | Phosphopantetheine-binding protein |
|  | *dfn*K | HU024_05630 | Cytochrome P450 |
|  | *dfn*L | HU024_05635 | Hydroxymethylglutaryl-CoA synthase family protein |
|  | *dfn*M | HU024_05640 | Enoyl-CoA hydratase-related protein |
| Fengycin synthesis | *fen*A | HU024_07395 | Non-ribosomal peptide synthetase |
|  | *fen*B | HU024_07400 | Non-ribosomal peptide synthetase |
|  | *fen*C | HU024_07405 | Non-ribosomal peptide synthetase |
|  | *fen*C2 | HU024_07410 | Non-ribosomal peptide synthetase |
|  | *fen*D | HU024_07415 | Non-ribosomal peptide synthase |
| Bacillaene synthesis | *bae*S | HU024_08100 | Cytochrome P450 |
|  | *bae*R | HU024_08105 | Thioesterase domain-containing protein |
|  | *bae*N | HU024_08110 | Non-ribosomal peptide synthetase |
|  | *bae*M | HU024_08115 | SDR family NAD(P)-dependent oxidoreductase |
|  | *bae*L | HU024_08120 | SDR family NAD(P)-dependent oxidoreductase |
|  | *bae*J | HU024_08125 | Non-ribosomal peptide synthetase |
|  | *bae*I | HU024_08130 | Enoyl-CoA hydratase-related protein |
|  | *bae*H | HU024_08135 | Enoyl-CoA hydratase/isomerase |
|  | *bae*G | HU024_08140 | Hydroxymethylglutaryl-CoA synthase family protein |
|  | *bae*F | HU024_08145 | Acyl carrier protein |
|  | *bae*E | HU024_08150 | ACP S-malonyltransferase |
|  | *bae*D | HU024_08155 | Acyltransferase domain-containing protein |
|  | *bae*C | HU024_08160 | ACP S-malonyltransferase |
|  | *bae*B | HU024_08165 | MBL fold metallo-hydrolase |
| Macrolactin synthesis | *mln*I | HU024_09400 | Serine hydrolase |
|  | *mln*H | HU024_09405 | Alpha/beta fold hydrolase |
|  | *mln*G | HU024_09410 | SDR family NAD(P)-dependent oxidoreductase |
|  | *mln*F | HU024_09415 | SDR family NAD(P)-dependent oxidoreductase |
|  | *mln*E | HU024_09420 | SDR family NAD(P)-dependent oxidoreductase |
|  | *mln*D | HU024_09425 | Type I polyketide synthase |
|  | *mln*C | HU024_09430 | Type I polyketide synthase |
|  | *mln*B | HU024_09435 | SDR family NAD(P)-dependent oxidoreductase |
| Surfactin synthesis | *sfp* | HU024_14835 | 4'-phosphopantetheinyl transferase superfamily protein |
|  | *srf*AD | HU024_14845 | Surfactin biosynthesis thioesterase SrfAD |
|  | *srf*AC | HU024_14850 | Surfactin non-ribosomal peptide synthetase SrfAC |
|  | *srf*AB | HU024_14855 | Surfactin non-ribosomal peptide synthetase SrfAB |
|  | *srf*AA | HU024_14860 | Surfactin non-ribosomal peptide synthetase SrfAA |
| Bacilysin synthesis | *bac*A | HU024_18285 | Bacilysin biosynthesis protein BacA |
|  | *bac*B | HU024_18290 | Cupin domain-containing protein |
|  | *bac*C | HU024_18295 | Dihydroanticapsin 7-dehydrogenase |
|  | *bac*D | HU024_18300 | ATP-grasp domain-containing protein |
|  | *bac*E | HU024_18305 | MFS transporter |
| Butirosin A / butirosin B synthesis | *btr*X | HU024_11805 | ABC transporter ATP-binding protein |
|  | *btr*W | HU024_11810 | ABC transporter ATP-binding protein |

**Table S3 CAZy notes family results and gene functional predictions**

| Class definition | Family | Genes count | CAZy activities |
| --- | --- | --- | --- |
| Glycoside hydrolases (GHs) | GH0 | 5 | Glycoside hydrolase |
|  | GH1 | 6 | β-Glucosidase (EC3.2.1.21); β-galactosidase (EC3.2.1.23); β-mannosidase (EC3.2.1.25); β-glucuronidase (EC3.2.1.31); β-xylosidase (EC3.2.1.37); β-D-fucosidase (EC 3.2.1.38); phlorizin hydrolase (EC3.2.1.62); exo-β-1,4-glucanase (EC3.2.1.74); 6-phospho-β-galactosidase (EC3.2.1.85); 6-phospho-β-glucosidase (EC3.2.1.86); strictosidine β-glucosidase (EC3.2.1.105); lactase (EC3.2.1.108); amygdalin β-glucosidase (EC3.2.1.117); prunasin β-glucosidase (EC3.2.1.118); vicianin hydrolase (EC3.2.1.119); raucaffricine β-glucosidase (EC3.2.1.125); thioglucosidase (EC3.2.1.147); β-primeverosidase (EC3.2.1.149); isoflavonoid 7-O-β-apiosyl-β-glucosidase (EC3.2.1.161); ABA-specific β-glucosidase (EC3.2.1.175); DIMBOA β-glucosidase (EC3.2.1.182); hydroxyisourate hydrolase (EC3.-.-.-); β-rutinosidase /α-L-rhamnose-(1,6)-β-D-glucosidase (EC3.2.1.-); protodioscin 26-O-Î²-D-glucosidase (EC3.2.1.186); α-L-arabinopyranosidase (EC3.2.1.-); isoflavone 7-O-glucosyl β-glucosidase (EC3.2.1.-) |
|  | GH3 | 1 | β-Glucosidase (EC3.2.1.21); xylan 1,4-β-xylosidase (EC3.2.1.37); β-glucosylceramidase (EC3.2.1.45); β-N-acetylhexosaminidase (EC3.2.1.52); α-L-arabinofuranosidase (EC3.2.1.55); glucan 1,4-β-glucosidase (EC3.2.1.74); isoprimeverose-producing oligoxyloglucan hydrolase (EC3.2.1.120); coniferin β-glucosidase (EC3.2.1.126); exo-1,3-1,4-glucanase (EC3.2.1.-); β-N-acetylglucosaminide phosphorylases (EC2.4.1.-); β-1,2-glucosidase (EC3.2.1.-); β-1,3-glucosidase (EC3.2.1.-); xyloglucan-specific exo-β-1,4-glucanase / exo-xyloglucanase (EC3.2.1.155); stevioside-β-1,2-glucosidase (EC3.2.1.-); lichenase / endo-β-1,3-1,4-glucanase (EC3.2.1.73); protodioscin 26-O-β-D-glucosidase (EC3.2.1.186); β-glucuronidase (EC3.2.1.31); avenacinase (EC3.2.1.-); tomatinase β-1,2-glucosidase (EC3.2.1.-) |
|  | GH4 | 5 | Maltose-6-phosphate glucosidase (EC3.2.1.122); α-glucosidase (EC3.2.1.20); α-galactosidase (EC3.2.1.22); 6-phospho-β-glucosidase (EC3.2.1.86); α-glucuronidase (EC3.2.1.139); α-galacturonase (EC3.2.1.67); palatinase (EC3.2.1.-) |
|  | GH5 | 2 | Endo-β-1,4-glucanase / cellulase (EC3.2.1.4); endo-β-1,4-xylanase (EC3.2.1.8); β-glucosidase (EC3.2.1.21); β-mannosidase (EC3.2.1.25); β-gluendo-β-1,4-glucanase / cellulase (EC3.2.1.4); endo-β-1,4-xylanase (EC3.2.1.8); β-glucosidase (EC3.2.1.21); β-mannosidase (EC3.2.1.25); β-glucosylceramidase (EC3.2.1.45); glucan β-1,3-glucosidase (EC3.2.1.58); exo-β-1,4-glucanase / cellodextrinase (EC3.2.1.74); glucan endo-1,6-β-glucosidase (EC3.2.1.75); mannan endo-β-1,4-mannosidase (EC3.2.1.78); cellulose β-1,4-cellobiosidase (EC3.2.1.91); steryl β-glucosidase (EC 3.2.1.104); endoglycoceramidase (EC3.2.1.123); β-primeverosidase (EC3.2.1.149); xyloglucan-specific endo-β-1,4-glucanase (EC3.2.1.151); endo-β-1,6-galactanase (EC3.2.1.164); β-1,3-mannanase (EC3.2.1.-); arabinoxylan-specific endo-β-1,4-xylanase (EC3.2.1.-); mannan transglycosylase (EC2.4.1.-); lichenase / endo-β-1,3-1,4-glucanase (EC3.2.1.73); β-glycosidase (EC3.2.1.-); endo-β-1,3-glucanase / laminarinase (EC3.2.1.39); β-N-acetylhexosaminidase (EC3.2.1.52); chitosanase (EC3.2.1.132); β-D-galactofuranosidase (EC3.2.1.146); β-galactosylceramidase (EC3.2.1.46); ; β-rutinosidase /α-L-rhamnose-(1,6)-β-D-glucosidase (EC3.2.1.-); α-L-arabinofuranosidase (EC3.2.1.55); glucomannan-specific endo-β-1,4-glucanase (EC3.2.1.-); hesperidin 6-O-α-L-rhamnosyl-β-glucosidase (EC3.2.1.168) |
|  | GH9 | 1 | Endoglucanase (EC3.2.1.4); endo-β-1,3(4)-glucanase / lichenase-laminarinase (EC3.2.1.6); lichenase / endo-β-1,3-1,4-glucanase (EC3.2.1.73); exo-β-1,4-glucanase / cellodextrinase (EC3.2.1.74); cellobiohydrolase (EC3.2.1.91); xyloglucan-specific endo-β-1,4-glucanase / endo-xyloglucanase (EC3.2.1.151); exo-β-glucosaminidase (EC3.2.1.165); endo-β-1,4-glucanase (xanthanase) (EC3.2.1.-) |
|  | GH11 | 1 | Endo-β-1,4-xylanase (EC3.2.1.8); exo-1,4-β-xylosidase (EC3.2.1.-) |
|  | GH13 | 15 | Oligosaccharide α-4-glucosyltransferase (EC2.4.1.161); palatinase (EC3.2.1.-); α-amylase (EC3.2.1.1); oligo-α-1,6-glucosidase (EC3.2.1.10); α-glucosidase (EC3.2.1.20); glucodextranase (EC3.2.1.70);isomaltulose synthase / sucrose isomerase / sucrose glucosylmutase (EC5.4.99.11) |
|  | GH16 | 1 | Xyloglucan:xyloglucosyltransferase (EC2.4.1.207); keratan-sulfate endo-1,4-β-galactosidase (EC3.2.1.103); endo-1,3-β-glucanase / laminarinxyloglucan:xyloglucosyltransferase (EC2.4.1.207); keratan-sulfate endo-1,4-β-galactosidase (EC3.2.1.103); endo-1,3-β-glucanase / laminarinase (EC3.2.1.39); endo-1,3(4)-β-glucanase (EC3.2.1.6); licheninase (EC3.2.1.73); β-agarase (EC3.2.1.81); κ-carrageenase (EC3.2.1.83); xyloglucanase (EC3.2.1.151); endo-β-1,3-galactanase (EC3.2.1.181); β-porphyranase (EC3.2.1.178); hyaluronidase (EC3.2.1.35); endo-β-1,4-galactosidase (EC3.2.1.-); chitin β-1,6-glucanosyltransferase (EC2.4.1.-); β-transglycosidase (EC2.4.1.-); β-glycosidase (EC3.2.1.-); β-carrageenase (EC3.2.1.-) |
|  | GH18 | 2 | Chitinase (EC3.2.1.14); lysozyme (EC3.2.1.17); endo-β-N-acetylglucosaminidase (EC3.2.1.96); peptidoglycan hydrolase with endo-β-N-acetylglucosaminidase specificity (EC3.2.1.-); Nod factor hydrolase (EC3.2.1.-); xylanase inhibitor; concanavalin B; narbonin; dΙ-N-acetylchitobiase / reducing-end exo-hexosaminidase (EC3.2.1.-); chitobiosidase (EC3.2.1.200) |
|  | GH23 | 5 | Lysozyme type G (EC3.2.1.17); peptidoglycan lyase (EC4.2.2.1) also known in the literature as peptidoglycan lytic transglycosylase; chitinase (EC3.2.1.14) |
|  | GH24 | 1 | Lysozyme (EC3.2.1.17) |
|  | GH25 | 1 | Lysozyme (EC3.2.1.17) |
|  | GH26 | 1 | β-Mannanase (EC3.2.1.78); exo-β-1,4-mannobiohydrolase (EC3.2.1.100); β-1,3-xylanase (EC3.2.1.32); lichenase / endo-β-1,3-1,4-glucanase (EC3.2.1.73); mannobiose-producing exo-β-mannanase (EC3.2.1.-) |
|  | GH28 | 3 | Polygalacturonase (EC3.2.1.15); exo-polygalacturonase (EC3.2.1.67); exo-polygalacturonosidase (EC3.2.1.82); rhamnogalacturonase (EC3.2.1.171); rhamnogalacturonan α-1,2-galacturonohydrolase (EC3.2.1.173); xylogalacturonan hydrolase (EC3.2.1.-) |
|  | GH30 | 2 | β-1,6-Glucosidase (EC3.2.1.-); endo-β-1,6-glucanase (EC3.2.1.75) |
|  | GH32 | 3 | Nvertase (EC3.2.1.26); endo-inulinase (EC3.2.1.7); β-2,6-fructan 6-levanbiohydrolase (EC3.2.1.64); endo-levanase (EC3.2.1.65); exo-inulinase (EC3.2.1.80); fructan β-(2,1)-fructosidase/1-exohydrolase (EC3.2.1.153); fructan β-(2,6)-fructosidase/6-exohydrolase (EC3.2.1.154); sucrose:sucrose 1-fructosyltransferase (EC2.4.1.99); fructan:fructan 1-fructosyltransferase (EC2.4.1.100); sucrose:fructan 6-fructosyltransferase (EC2.4.1.10); fructan:fructan 6G-fructosyltransferase (EC 2.4.1.243); levan fructosyltransferase (EC2.4.1.-); sucrose:sucrose 6-fructosyltransferase (6-SST) (EC2.4.1.-); cycloinulo-oligosaccharide fructanotransferase (EC2.4.1.-) |
|  | GH38 | 1 | α-Mannosidase (EC3.2.1.24); mannosyl-oligosaccharide α-1,2-mannosidase (EC3.2.1.113); mannosyl-oligosaccharide α-1,3-1,6-mannosidase (EC3.2.1.114); mannosyl-oligosaccharide α-1,3-mannosidase (EC3.2.1.207); mannosyl-oligosaccharide α-1,6-mannosidase / exo-α-1,6-mannosidase (EC3.2.1.163) |
|  | GH43 | 4 | β-Xylosidase (EC3.2.1.37); α-L-arabinofuranosidase (EC3.2.1.55); xylanase (EC3.2.1.8); α-1,2-L-arabinofuranosidase (EC3.2.1.-); exo-α-1,5-L-arabinofuranosidase (EC3.2.1.-); [inverting] exo-α-1,5-L-arabinanase (EC3.2.1.-); β-1,3-xylosidase (EC3.2.1.72); [inverting] exo-α-1,5-L-arabinanase (EC3.2.1.-); [inverting] endo-α-1,5-L-arabinanase (EC3.2.1.99); exo-β-1,3-galactanase (EC3.2.1.145); β-D-galactofuranosidase (EC3.2.1.146) |
|  | GH46 | 1 | Chitosanase (EC3.2.1.132) |
|  | GH51 | 2 | Endoglucanase (EC3.2.1.4); endo-β-1,4-xylanase (EC3.2.1.8); β-xylosidase (EC3.2.1.37); α-L-arabinofuranosidase (EC3.2.1.55); cellobiohydrolase (EC3.2.1.91) |
|  | GH53 | 1 | Endo-β-1,4-galactanase (EC3.2.1.89). |
|  | GH65 | 1 | α,α-Trehalase (EC 3.2.1.28); maltose phosphorylase (EC2.4.1.8); trehalose phosphorylase (EC2.4.1.64); kojibiose phosphorylase (EC2.4.1.230); trehalose-6-phosphate phosphorylase (EC2.4.1.216); nigerose phosphorylase (EC2.4.1.279); 3-O-α-glucopyranosyl-L-rhamnose phosphorylase (EC2.4.1.282); 1,2-α-glucosylglycerol phosphorylase (EC2.4.1.332); α-glucosyl-1,2-β-galactosyl-L-hydroxylysine α-glucosidase (EC3.2.1.107); 1,3-α-oligoglucan phosphorylase (EC2.4.1.334); α-1,2-glucosidase (EC3.2.1.-); α-glucan phosphorylase (EC2.4.1.-); kojibiose glucohydrolase (α-1,2-glucosidase) (configuration inverting) (EC3.2.1.216); branched-dextran exo-1,2-Î±-glucosidase (EC3.2.1.115); |
|  | GH68 | 1 | Levansucrase (EC2.4.1.10); β-fructofuranosidase (EC3.2.1.26); inulosucrase (EC2.4.1.9). |
|  | GH73 | 2 | Lysozyme (EC3.2.1.17); mannosyl-glycoprotein endo-β-N-acetylglucosaminidase (EC3.2.1.96); peptidoglycan hydrolase with endo-β-N-acetylglucosaminidase specificity (EC3.2.1.-) |
|  | GH84 | 1 | N-acetyl β-glucosaminidase (EC3.2.1.52); hyaluronidase (EC3.2.1.35); [protein]-3-O-(GlcNAc)-L-Ser/Thr β-N-acetylglucosaminidase (EC3.2.1.169) |
|  | GH101 | 1 | Endo-α-N-acetylgalactosaminidase (EC3.2.1.97) |
|  | GH109 | 2 | α-N-acetylgalactosaminidase (EC3.2.1.49); β-N-acetylhexosaminidase (EC3.2.1.52) |
|  | GH126 | 2 | α-Amylase (EC3.2.1.-) |
|  | GH171 | 1 | Peptidoglycan β-N-acetylmuramidase (EC3.2.1.92) |
| Glycosyl transferases (GTs) | GT0 | 5 | Glycosyl transferases |
|  | GT1 | 11 | UDP-glucuronosyltransferase (EC2.4.1.17); zeatin O-β-xylosyltransferase (EC2.4.2.40); 2-hydroxyacylsphingosine 1-β-galactosyltransferase (EC2.4.1.45); N-acylsphingosine galactosyltransferase (EC2.4.1.47); flavonol 3-O-glucosyltransferase (EC2.4.1.91); anthocyanidin 3-O-glucosyltransferase (EC2.4.1.115); sinapate 1-glucosyltransferase (EC2.4.1.120); indole-3-acetate β-glucosyltransferase (EC2.4.1.121); flavonol L-rhamnosyltransferase (EC 2.4.1.159); sterol glucosyltransferase (EC2.4.1.173); UDP-Glc: 4-hydroxybenzoate 4-O-β-glucosyltransferase (EC2.4.1.194); zeatin O-β-glucosyltransferase (EC2.4.1.203); limonoid glucosyltransferase (EC2.4.1.210); UDP-GlcA: baicalein 7-O-β-glucuronosyltransferase (EC2.4.1.253); UDP-Glc: chalcone 4'-O-β-glucosyltransferase (EC2.4.1.286); ecdysteroid UDP-glucosyltransferase (EC2.4.1.-); salicylic acid β-glucosyltransferase (EC2.4.1.-); anthocyanin 3-O-galactosyltransferase (EC2.4.1.-); anthocyanin 5-O-glucosyltransferase (EC2.4.1.-); dTDP-β-2-deoxy-L-fucose: α-L-2-deoxyfucosyltransferase (EC2.4.1.-); UDP-β-L-rhamnose: α-L-rhamnosyltransferase (EC2.4.1.-); zeaxanthin glucosyltransferase (EC2.4.1.-); UDP-Glc: flavone-6-C-glucosytransferase (EC2.4.1.-); UDP-Glc: cinnamate β-glucosyltransferase (EC2.4.1.177); UDP-Glc: hydroxycinnamic acid O-β-glucosyltransferase (EC2.4.1.-); UDP-Glc: cinnamoyl O-β-glucosyltransferase (EC2.4.1.-); UDP-Arap: flavone-C-arabinosytransferase (EC2.4.1.-); [inverting] UDP-Glc: ginsenoside 3-O-glucosyltransferase (EC2.4.1.364); [inverting] UDP-Glc: 3-O-glucosyl-protopanaxadiol-type ginsenoside 2''-O-glucosyltransferase (EC2.4.1.365); UDP-Glc: p-hydroxymandelonitrile-O-glucosyltransferase (EC2.4.1.85) |
|  | GT2 | 40 | Cellulose synthase (EC2.4.1.12); chitin synthase (EC2.4.1.16); dolichyl-phosphate β-D-mannosyltransferase (EC2.4.1.83); dolichyl-phosphate β-glucosyltransferase (EC2.4.1.117); N-acetylglucosaminyltransferase (EC2.4.1.-); N-acetylgalactosaminyltransferase (EC2.4.1.-); hyaluronan synthase (EC2.4.1.212); chitin oligosaccharide synthase (EC2.4.1.-); β-1,3-glucan synthase (EC2.4.1.34); β-1,4-mannan synthase (EC2.4.1.-); β-mannosylphosphodecaprenol-mannooligosaccharide α-1,6-mannosyltransferase (EC2.4.1.199); UDP-Galf: rhamnopyranosyl-N-acetylglucosaminyl-PP-decaprenol β-1,4/1,5-galactofuranosyltransferase (EC2.4.1.287); UDP-Galf: galactofuranosyl-galactofuranosyl-rhamnosyl-N-acetylglucosaminyl-PP-decaprenol β-1,5/1,6-galactofuranosyltransferase (EC2.4.1.288); dTDP-L-Rha: N-acetylglucosaminyl-PP-decaprenol α-1,3-L-rhamnosyltransferase (EC2.4.1.289); alternating β-1,3/4-N-acetylmannan synthase (2.4.1.-); UDP-GlcA: N-acetylglucosaminyl-proteoglycan β-1,4-glucuronosyltransferase (EC2.4.1.225); [inverting] UDP-Glc: glycocin S-β-glucosyltransferase (EC2.4.1.-); [inverting] UDP-Glc: protein O-β-glucosyltransferase (EC 2.4.1.-) |
|  | GT4 | 11 | Sucrose synthase (EC2.4.1.13); sucrose-phosphate synthase (EC2.4.1.14); α-glucosyltransferase (EC4.1.52); lipopolysaccharide N-acetylglucosaminyltransferase (EC2.4.1.56); phosphatidylinositol α-mannosyltransferase (EC2.4.1.57); GDP-Man: Man1GlcNAc2-PP-dolichol α-1,3-mannosyltransferase (EC2.4.1.132); GDP-Man: Man3GlcNAc2-PP-dolichol/Man4GlcNAc2-PP-dolichol α-1,2-mannosyltransferase (EC2.4.1.131); digalactosyldiacylglycerol synthase (EC2.4.1.141); 1,2-diacylglycerol 3-glucosyltransferase (EC2.4.1.157); diglucosyl diacylglycerol synthase (EC2.4.1.208); trehalose phosphorylase (EC2.4.1.231); NDP-Glc: α-glucose α-glucosyltransferase / α,α-trehalose synthase (EC2.4.1.245); GDP-Man: Man2GlcNAc2-PP-dolichol α-1,6-mannosyltransferase (EC2.4.1.257); UDP-GlcNAc: 2-deoxystreptamine α-N-acetylglucosaminyltransferase (EC2.4.1.283); UDP-GlcNAc: ribostamycin α-N-acetylglucosaminyltransferase (EC2.4.1.285); UDP-Gal α-galactosyltransferase (EC2.4.1.-); UDP-Xyl α-xylosyltransferase (EC2.4.2.-); UDP-GlcA α-glucuronyltransferase (EC2.4.1.-); UDP-Glc α-glucosyltransferase (EC2.4.1.-); UDP-GalNAc: GalNAc-PP-Und α-1,3-N-acetylgalactosaminyltransferase (EC2.4.1.306); UDP-GalNAc: N,N'-diacetylbacillosaminyl-PP-Und α-1,3-N-acetylgalactosaminyltransferase (EC2.4.1.290); ADP-dependent α-maltose-1-phosphate synthase (2.4.1.342);UDP-GlcNAc: polypeptide α-N-acetylglucosaminyltransferase (EC2.4.1.-); UDP-GlcNAc: α-N-acetylglucosaminyltransferase (EC2.4.1.-) |
|  | GT8 | 1 | Lipopolysaccharide α-1,3-galactosyltransferase (EC2.4.1.44); UDP-Glc: (glucosyl)lipopolysaccharide α-1,2-glucosyltransferase (EC2.4.1.-); lipopolysaccharide glucosyltransferase 1 (EC2.4.1.58); glycogenin glucosyltransferase (EC2.4.1.186); inositol 1-α-galactosyltransferase (galactinol synthase) (EC2.4.1.123); homogalacturonan α-1,4-galacturonosyltransferase (EC2.4.1.43); UDP-GlcA: xylan α-glucuronyltransferase (EC2.4.1.-); UDP-Gal:glucoside α-1,3-galactosyltransferase (EC2.4.1.-) |
|  | GT26 | 1 | UDP-ManNAcA: β-N-acetyl mannosaminuronyltransferase (EC2.4.1.-); UDP-ManNAc: β-N-acetyl-mannosaminyltransferase (EC2.4.1.-); UDP-Glc: β-1,4-glucosyltransferase (EC2.4.1.-); UDP-Gal: β-1,4-galactosyltransferase (EC 2.4.1.-) |
|  | GT28 | 2 | 1,2-diacylglycerol 3-β-galactosyltransferase (EC2.4.1.46); 1,2-diacylglycerol 3-β-glucosyltransferase (EC2.4.1.157); UDP-GlcNAc: Und-PP-MurAc-pentapeptide β-N-acetylglucosaminyltransferase (EC2.4.1.227); digalactosyldiacylglycerol synthase (EC2.4.1.241) |
|  | GT30 | 1 | CMP-β-KDO: α-3-deoxy-D-manno-octulosonic-acid (KDO) transferase (EC2.4.99.-). |
|  | GT51 | 5 | Murein polymerase (EC2.4.1.129) |
|  | GT58 | 1 | Dol-P-Man: Man5GlcNAc2-PP-Dol α-1,3-mannosyltransferase (EC2.4.1.258) |
|  | GT83 | 2 | Undecaprenyl phosphate-α-L-Ara4N: 4-amino-4-deoxy-β-L-arabinosyltransferase (EC2.4.2.43); dodecaprenyl phosphate-β-galacturonic acid: lipopolysaccharide core α-galacturonosyl transferase (EC2.4.1.-) |
| Polysaccharide lyases (PLs) | PL1_6 | 1 | Pectate lyase (EC4.2.2.2) |
|  | PL1_8 | 1 | Pectin lyase (EC4.2.2.10) |
|  | PL9_2 | 1 | Pectate lyase (EC4.2.2.2); exopolygalacturonate lyase (EC4.2.2.9); thiopeptidoglycan lyase (EC4.2.2.-); rhamnogalacturonan endolyase (EC4.2.2.23) |
| Carbohydrate esterases (CEs) | CE14 | 3 | N-acetyl-1-D-myo-inosityl-2-amino-2-deoxy-α-D-glucopyranoside deacetylase (EC3.5.1.89); diacetylchitobiose deacetylase (EC3.5.1.-); mycothiol S-conjugate amidase (EC3.5.1.-) |
|  | CE0 | 1 | Acetyl xylan esterase (EC3.1.1.72). |
|  | CE1 | 3 | Acetyl xylan esterase (EC3.1.1.72); cinnamoyl esterase (EC3.1.1.-); feruloyl esterase (EC3.1.1.73); carboxylesterase (EC3.1.1.1); S-formylglutathione hydrolase (EC3.1.2.12); diacylglycerol O-acyltransferase (EC2.3.1.20); trehalose 6-O-mycolyltransferase (EC2.3.1.122) |
|  | CE4 | 8 | Acetyl xylan esterase (EC3.1.1.72); chitin deacetylase (EC3.5.1.41); chitooligosaccharide deacetylase (EC3.5.1.-); peptidoglycan GlcNAc deacetylase (EC3.5.1.-); peptidoglycan N-acetylmuramic acid deacetylase (EC3.5.1.-). |
|  | CE7 | 1 | Acetyl xylan esterase (EC3.1.1.72); cephalosporin-C deacetylase (EC3.1.1.41). |
|  | CE8 | 1 | Pectin methylesterase (EC3.1.1.11). |
|  | CE9 | 1 | N-acetylglucosamine 6-phosphate deacetylase (EC3.5.1.25); N-acetylgalactosamine 6-phosphate deacetylase (EC3.5.1.80) |
|  | CE12 | 1 | Pectin acetylesterase (EC3.1.1.-); rhamnogalacturonan acetylesterase (EC3.1.1.-); acetyl xylan esterase (EC3.1.1.72) |
| Carbohydrate-binding modules (CBMs) | CBM3 | 1 | Modules of approx. 150 residues found in bacterial enzymes. The cellulose-binding function has been demonstrated in many cases. In one instance binding to chitin has been reported. |
|  | CBM6 | 2 | Modules of approx. 120 residues. The cellulose-binding function has been demonstrated in one case on amorphous cellulose and β-1,4-xylan. Some of these modules also bind β-1,3-glucan, β-1,3-1,4-glucan, and β-1,4-glucan. |
|  | CBM34 | 1 | Modules of approx. 120 residues. Granular starch-binding function has been demonstrated in the case of thermoactinomyces vulgaris R-47 α-amylase 1 (TVAI). |
|  | CBM50 | 21 | Modules of approx. 50 residues found attached to various enzymes from families GH18, GH19, GH23, GH24, GH25 and GH73, enzymes cleaving either chitin or peptidoglycan. Binding to chitopentaose demonstrated in the case of Pteris ryukyuensis chitinase A [Ohnuma T et al. (2008) J. Biol. Chem. 283:5178-87 (PMID: 18083709)]. CBM50 modules are also found in a multitude of other enzymes targeting the petidoglycan such as peptidases and amidases. These enzymes are not reported in the list below. |
| Auxiliary activities (AAs) | AA1 | 1 | Laccase / p-diphenol:oxygen oxidoreductase / ferroxidase (EC1.10.3.2); ferroxidase (EC1.10.3.-); Laccase-like multicopper oxidase (EC1.10.3.-) |
|  | AA4 | 1 | vanillyl-alcohol oxidase (EC1.1.3.38) |
|  | AA6 | 1 | 1,4-benzoquinone reductase (EC1.6.5.6) |
|  | AA7 | 2 | glucooligosaccharide oxidase (EC1.1.3.-); chitooligosaccharide oxidase (EC1.1.3.-); cellooligosaccharide dehydrogenase (EC1.1.99.-) |
|  | AA10 | 1 | AA10 (formerly CBM33) proteins are copper-dependent lytic polysaccharide monooxygenases (LPMOs); some proteins have been shown to act on chitin, others on cellulose; lytic cellulose monooxygenase (C1-hydroxylating) (EC1.14.99.54); lytic cellulose monooxygenase (C4-dehydrogenating) (EC1.14.99.56); lytic chitin monooxygenase (EC1.14.99.53); lytic xylan monooxygenase / xylan oxidase (glycosidic bond-cleaving) (EC1.14.99.-) |
